# A novel protein B2URF3 from *Akkermansia muciniphila* increased by intermittent fasting alleviates vascular calcification

**DOI:** 10.1186/s12951-025-03948-0

**Published:** 2026-01-07

**Authors:** Shi-Yu Zeng, Jiang-Hua Liu, Ying-Ying Xiang, Zhao-Lin Zeng, Zhi-Bo Zhao, Jie zheng, Yi-Fu Liu, Zhi-Rou Lin, Yi-Yi Wang, Chun-Gu Hong, Ling Jin, Guo-Qiang Zhu, Yi-Wei Liu, Xin Wang, Xiao-Xue Li, Zhe Guan, Zhen-Xing Wang, Ting Sun, Hui Xie, Jiang-Hua Liu

**Affiliations:** 1 Department of Metabolism and Endocrinology, The First Affiliated Hospital, Hengyang Medical School, University of South China, Hengyang, 421001 Hunan China; 2 Department of Ultrasound Medicine, The First Affiliated Hospital, Hengyang Medical School, University of South China, Hengyang, 421002 China; 3 Department of Orthopedics, Movement System Injury and Repair Research Center, Xiangya Hospital, Central South University, Changsha, 410008 Hunan China; 4 National Clinical Research Center for Geriatric Disorders, Xiangya Hospital, Central South University, Changsha, 410008 Hunan China; 5 Hunan Key Laboratory of Angmedicine, Changsha, 410008 Hunan China; 6 Department of Orthopedics, The First Affiliated Hospital, Hengyang Medical School, University of South China, Hengyang, 421001 Hunan China; 7 Hunan Diabetes Clinical Medical Research Center, Hengyang, 421001 Hunan China; 8 Department of Urology, The Second Affiliated Hospital, Hengyang Medical School, University of South China, Hengyang, Hunan China; 9 Department of Cardiovascular Medicine, The First Affiliated Hospital of Chongqing Medical University, Chongqing, 400016 China; 10 Department of Urology, The First Affiliated Hospital of Nanchang University, Nanchang, 330000 Jiangxi China

**Keywords:** Intermittent fasting, Gut-vascular axis, Akkermansia muciniphila, Extracellular vesicles, Vascular calcification

## Abstract

Vascular calcification (VC) is a major contributor to cardiovascular morbidity and mortality, yet effective therapies are lacking. Here, we show that alternate-day intermittent fasting (IF1:1) attenuates vitamin D-induced VC in mice, whereas a 5:2 regimen is ineffective. The protective effect of IF1:1 is gut microbiota-dependent, particularly through enrichment of *Akkermansia muciniphila* (*Akk*). Microbiota-derived extracellular vesicles (EVs) function as nano-scale mediators that bypass the spatiotemporal constraints of bacterial survival to facilitate long-distance communication with host cells, providing a crucial pathway for downstream mechanistic investigation. *Akk*-derived EVs (*Akk*-EVs) are internalized by vascular smooth muscle cells (VSMCs), suppressing osteogenic differentiation and calcification in vitro and in vivo. Proteomic analysis identified B2URF3 as a highly enriched functional protein in *Akk*-EVs and *Akk*, which interacts with Aldehyde Dehydrogenase 1 Family Member B1 (ALDH1B1) to inhibit VSMC osteogenic transdifferentiation. Clinically, reduced fecal *Akk* abundance and lower serum B2URF3 levels were observed in patients with coronary calcification. These findings define a gut-vascular axis by which IF1:1 mitigates VC and nominate *Akk*-EVs and B2URF3 as potential therapeutic targets and biomarkers.

**Graphical abstract:**

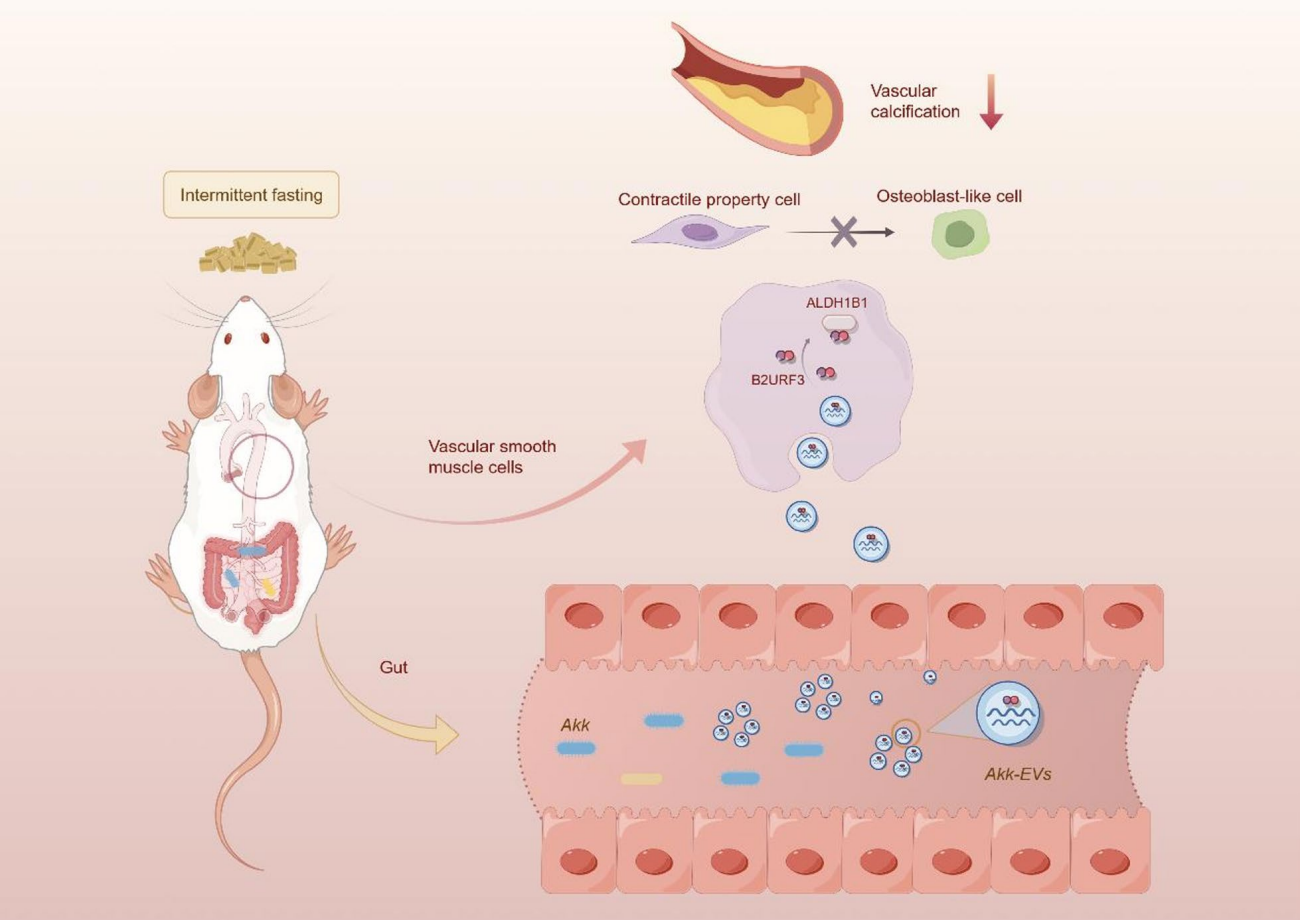

**Supplementary Information:**

The online version contains supplementary material available at 10.1186/s12951-025-03948-0.

## Introduction

Vascular calcification (VC) is a pathological process characterized by the deposition of calcium phosphate crystals in the arterial wall, which is strongly associated with increased risk of cardiovascular disease [1]. Therefore, developing effective therapeutic strategies to prevent or treat this condition is of great importance. Intermittent fasting (IF), an emerging dietary strategy that involves cycling between periods of fasting and non-fasting including alternate day fasting (IF1:1) and 5:2 intermittent fasting (IF5:2) [2], has gained considerable attention in recent years due to its potential effects on metabolism, inflammation and cardiovascular health [3, 4]. Several studies have reported beneficial effects of IF on weight loss [4], insulin sensitivity [5], lipid profile [6], and blood pressure [7], as well as a reduction in markers of inflammation and oxidative stress [8]. However, whether IF can protect aorta from calcification remains to be elucidated.

Gut microbiota (GM) is a diverse ecosystem comprising of symbiotic and mutualistic bacteria that are believed to interact with most organs of the host [9]. It is an essential physiological factor for promoting development and maintaining internal balance. Dietary changes have a significant impact on GM, and preclinical and clinical studies have shown that IF has a positive regulatory effect on GM. IF provides a period of “intestinal rest” that positively affects host health by improving GM composition, abundance, diversity, intestinal barrier function, immune and inflammatory responses, and production of derived metabolites such as short-chain fatty acids. The abundance of several beneficial bacteria, including *Lactobacillus* and *Bifidobacterium*, was significantly increased after IF intervention, and *Odoribacter*, which is negatively associated with blood pressure, also proliferated [10, 11]. Ramadan fasting as an IF protocol promotes positive changes in GM, including elevated abundance of *Akkermansia muciniphila* (*Akk*), *Bacteroides fragilis*, *Bacillus mimicus* and *Lactobacillus butyricus*-producing family, which are thought to be associated with improved health indicators and reduced disease progression during Ramadan [10, 12].

Communication between GM and host can be achieved through various pathways such as metabolic, immune, endocrine and neuronal. In recent years, GM-secreted extracellular versicles (EVs) have also been reported to be involved in GM-host communication. EV as a cellular secretory granule recognized from eukaryotes, bacteria and archaea mostly perform their functions in an autocrine or paracrine manner. Bacterial EVs can be loaded with a variety of bioactive molecules, including proteins, RNA and DNA, to regulate important biological functions which have an overall impact on host health. EVs originating from specific bacteria can exert different biological effects by eliciting different physiological responses. For example, EVs of *Pseudomonas panamensis*-derived from an aging population can cause cognitive impairment by crossing the blood-brain barrier [13]. In contrast, *Akk*-EVs were found to alleviate high-fat diet-induced obesity and insulin resistance in mice [14]. Meanwhile, our preliminary study found that *Akk*-EVs can exert anti-osteoporotic effects by promoting bone formation in osteoblasts and inhibiting bone resorption in osteoclasts [15].

Here, we investigated whether different IF regimens could protect against vitamin D (VD)-induced VC in mice by modulating beneficial gut microbiota. We further evaluated the effects of transplantation of the candidate bacterium *Akk* and its extracellular vesicles (*Akk*-EVs) on VC progression. Proteomic analysis identified B2URF3 as the most abundant protein in *Akk*-EVs, which recapitulated the anti-calcification effects of Akk and Akk-EVs. Immunoprecipitation–mass spectrometry (IP-MS) revealed that B2URF3 interacts with Aldehyde Dehydrogenase 1 Family Member B1(ALDH1B1) to inhibit osteogenic transdifferentiation of VSMCs. These findings provide mechanistic evidence for the protective effects of IF on VC and highlight *Akk* and its EVs as promising therapeutic candidates for clinical intervention.

## Methods

### Human samples

Aortic calcification was diagnosed by experienced clinical radiologists through computed tomography scanning. Exclusion criteria refer to patients who have taken antibiotics, probiotics, biologics and laxatives within the last 2 months or who have tumors. The clinical study was strictly conducted under the principles of the Declaration of Helsinki. Human fecal samples were collected at the First Affiliated Hospital of Nanchang University with approval from the Ethics Committee of the First Affiliated Hospital of Nanchang University. Serum samples were collected at the First Affiliated Hospital of University of South China with approval from the Ethics Committee of the First Affiliated Hospital of University of South China. All patients signed a written informed consent form.

### Animals and treatment

All experimental animals were conducted in accordance with the Chinese animal protection law and the National Institutes of Health (NIH) Guide for the Care and Use of Laboratory Animals. The experimental animal protocols were reviewed and approved by the Ethics Committee of South China University. 8-week-old C57BL/6 male mice were used in this study. To evaluate the effect of different intermittent fasting patterns on vascular calcification. We randomized C57BL/6J into four groups: ad libitum (AL) + PBS, AL + VD, IF1:1 + VD, and IF5:2 + VD. In this study, AL indicates unrestricted food access; IF1:1 refers to alternating 24-hour fasting and feeding; and IF5:2 represents 5 days of feeding followed by 2 days of fasting. After 28 days of feeding, the first group of mice was given PBS intraperitoneally as a blank control group, and the remaining three groups were given VD (750 U/g/d) intraperitoneally for 4 consecutive days to establish VC model, and after 10 days the mice were sacrificed and their aorta, serum, and feces were collected for further testing. To evaluate whether GM was involved in the alleviation of vascular calcification during intermittent fasting, we cleared the GM by adding a broad-spectrum antibiotic cocktail to the drinking water, which consisted of vancomycin (0.5 mg/ml), ampicillin (1 mg/ml), neomycin sulfate (1 mg/ml), and metronidazole (1 mg/ml). Then, feces collected from IF1:1 and AL fed mice were resuspended in PBS and centrifuged at 500 g for 1 min to collect the supernatant. All mice received intraperitoneal injections of VD for 4 consecutive days and were sacrificed ten days later. The microbiota-depleted recipients were administered supernatant orally twice a week for 4 weeks. To assess the role of *Akk* and its EVs in VC, we administered 3*10^8 CFUs *Akk* and 200 µg *Akk*-EVs to mice by gavage intervention twice a week for 4 weeks. To investigate the impact of candidate proteins in *Akk*-EVs on VC, mice were orally administered with *Akk*-EVs (200 µg in 200 µl of PBS for every mouse), several recombinant candidate proteins (75 µg in 200 µl of PBS for every mouse) or an equal volume of PBS twice a week for 4 weeks. Based on precedent of systemic administration of microbiota-derived EVs [16, 17], we delivered *Akk*-EVs (50 µg in 100 µl of PBS for every mouse) via tail-vein injection to ensure accurate and reproducible systemic exposure. Mice were euthanized by a single intraperitoneal injection of sodium pentobarbital (200 mg/kg), the aorta of mice was collected for Alizarin Red S staining (ARS) and calcium content assay.

### Cell culture

Human VSMCs (ATCC, CRL-1999) were cultured in Dulbecco’s Modified Eagle Medium/Nutrient Mixture F-12 containing 10% fetal bovine serum (FBS) and 1% penicillin–streptomycin, and cells at passages 3 to 8 were used for subsequent experiments. To induce VSMC calcification,

A*kk*-EVs and several candidate proteins were added to the osteogenic culture medium (HUXMX-90021, Cyagen Biosciences) during cell intervention. The mRNA expression levels and protein expression of osteogenic related factors were measured at 4 and 7 days after induction, respectively. ALP staining and ARS staining were performed at 5 and 15 days after induction, respectively.

### Alkaline phosphatase (ALP) staining

VSMCs were taken out of the growth medium and rinsed three times with phosphate-buffered saline (PBS). Subsequently, the VSMCs were fixed with a 4% solution of paraformaldehyde at room temperature for 20 min, followed by another three rinses with PBS. In the end, the VSMCs were treated with ALP staining solution (catalog number C3206, produced by Beyotime Biotechnology, Shanghai, China) at room temperature in the dark for a period ranging from 2 to 24 h.

### Determination of calcium content

The aortas underwent decalcification in 5% hydrochloric acid (HCl) at 4 °C for 48 h, after which the supernatant was harvested for calcium concentration analysis using a Calcium Ion Assay Kit (Beyotime Biotechnology), following the guidelines provided by the manufacturer. Protein concentration of aortic tissue was measured using the BCA assay (Elabscience) to normalize for calcium content.

### Von Kossa staining

Thoracic aortas were fixed in 4% paraformaldehyde (PFA) overnight at 4℃, followed by rinsing with distilled water twice. After dehydration, the paraffin-embedded aortic samples were sectioned into 5 μm-thick slices and prepared for von Kossa staining according to the manufacturer’s instructions (Solarbio).

### Alizarin red S (ARS) staining

For staining whole-mount aortas, the dissected aortas were immersed in 95% ethanol for 24 h at 37 °C. The staining solution was created by blending 0.5 mL of 0.1% alizarin red with 50 mL of 1% potassium hydroxide. The aortas were left to stain at room temperature throughout the night. Prior to imaging, they were rinsed with 2% potassium hydroxide twice.

For staining thoracic aortic sections, the 5-micrometer thick sections were first rehydrated and then treated with alizarin red. In the case of staining for VSMCs, these cells were plated into 48-well plates and subjected to the specific experimental conditions before being fixed for 20 min. Following two PBS washes, the calcium deposits within the VSMCs were highlighted with ARS for 2–5 min. The resulting images were captured using a light microscope.

### Immunofluorescence staining

*VSMCs.* VSMCs were seeded onto coverslips in 24-well plates and incubated overnight. After the intervention, cells were fixed with 4% PFA for 20 min and permeabilized with 0.3% Triton X-100 for 30 min at room temperature. Cells were then blocked with 5% bovine serum albumin for 1 h and incubated with targeted primary antibodies overnight at 4 °C. Cells were then incubated with secondary antibodies for 1 h at room temperature in the dark. Cell nuclei were stained with DAPI for 5 min and subsequently visualized using a fluorescence microscope.

*Aortic tissue*. Aortic specimens were fixed with 4% PFA for 24 h, immersed in 30% sucrose solution overnight, embedded in OCT, and finally made into 5 μm thick aortic sections. After the slides were rehydrated, they were permeabilized with 0.5% Triton X, blocked with 5% bovine serum albumin for 1 h, incubated with primary antibody at 4 °C overnight, and then incubated with secondary antibody at room temperature in the dark for 1 h. The nuclei were stained with DAPI, and then photographed using a fluorescence microscope.

### Culture of bacteria and isolation of bacterial EVs

*Akk* (ATCC, BAA-835) was grown anaerobically in BHI broth comprising 0.05% L cysteine–HCl (Sigma-Aldrich). Bacterial concentration is calculated by bacterial turbidity meter or by counting the number of colony-forming units of bacteria in BHI solid medium. After 4 days of incubation, the BHI medium was centrifuged at 4000xg for 30 min at 4 °C and the resulting precipitate was the desired bacteria. The supernatant was filtered by a 0.22 μm filter (100kD, Millipore, Billerica, USA) to remove the residual bacteria, then the resulting supernatant was concentrated 500 fold through an ultrafiltration tube (Millipore) by centrifugation at 4000 xg and 4 °C. According to the previous experimental protocol [15, 18], density gradient centrifugation was used to isolate bacterial EVs. The exosomal protein contents were determined using the BCA protein assay kit. The morphologies and diameters of *Akk*-EVs were evaluated using a transmission electron microscope (Hitachi H-7650; Tokyo, Japan) and nanoparticle tracking analysis (Particle Metrix, Meerbusch, Germany).

### EVs uptake detection

EVs were labeled with Dil dye and incubated for 30 min at 37 °C in a cell incubator protected from light. Unbound Dil dye was removed by using ultrafiltration centrifuge tubes (10 kD, Millipore). Labeled EVs were incubated with VSMCs for 12 h. After washing with PBS for 2 times, the above VSMCs were fixed by 4% paraformaldehyde and then nuclei were stained with DAPI. Fluorescence microscopy was used to acquire images.

### qRT-PCR analysis

To evaluate the relative *Akk* abundance in fecal, fecal DNA was extracted according to the proposal of TIANamp Stool DNA kit (TIANGEN, DP328) and amplified using qRT-PCR with *Akk* primers (forward, 5′-CCTTGCGGTTGGCTTCAGAT-3′, and reverse,5′-CAGCACGTGAAG GTGGGGAC-3′).

To assess the expression of osteogenic and contractile genes, RNA was extracted using Ultrapure RNA Kit (Cowin Bio, CW0581) according to the manufacturer’s instruction. The extracted RNA was reverse transcribed using cDNA Synthesis SuperMix (NovoScript, E044). Then, qRT-PCR was performed using SYBR Green qPCR Master Mix (Bimake, B21202). The sequences of RNAs were as follows: *human-Runx2*: forward, 5’- TGGTTACTGTCATGGCGGGTA-3’, and reverse, 5’- TCTCAGATCGTTGAACCTTGCTA-3’; *human-Ocn*: 5’- CACTCCTCGCCCTATTGGC-3’, and reverse, 5’- CCCTCCTGCTTGGACACAAAG − 3’; *human-Alpl*: forward, 5’- ACTGGTACTCAGACAACGAGAT-3’, and reverse, 5’- ACGTCAATGTCCCTGATGTTATG-3’; *human-αSMA*༚forward, 5’- TGCCAACAACGTCATGTCG − 3’, and reverse, 5’- CAGCGCGGTGATCTCTTTCT − 3’; *human- SM22α*༚forward, 5’- CCGTGGAGATCCCAACTGG − 3’, and reverse, 5’- CCATCTGAAGGCCAATGACAT − 3’; *human-Timm50*: forward, 5’- AGGGTCCCAGCTATGCCAA-3’, and reverse, 5’-TCCACCGGGTTGTTTCCAAAG-3’; human-Aldh1b1:forward,5’- CCCATTCTGAACCCAGACATC-3’, and reverse, 5’- AATGACCTCCCCGGTGGTA-3’.

###  16 s rRNA gene sequencing

DNA was extracted from the stool, and the V3-V4 region of the 16 S rRNA gene was amplified via PCR. The uniqueness and specificity of the PCR products were verified using agarose gel electrophoresis. Unique tag sequences were then attached to each sample, and these tags were incorporated into the library’s end via PCR. The amplified products were purified using magnetic beads for nucleic acid purification, yielding the initial sample libraries. Based on the preliminary quantification from agarose gel electrophoresis, the sample libraries with their specific index tags were appropriately diluted, and their concentrations were accurately measured with Qubit. The samples were pooled according to the sequencing requirements of each sample type. The size of the sequencing library’s insert fragments was confirmed using the Agilent 2100 Bioanalyzer to ensure there was no specific amplification in the 120–200 bp range, and the concentration of the sequencing libraries was precisely quantified. The library was ultimately sequenced on the NovaSeq 6000 platform using the SP-Xp (PE250) paired-end sequencing approach.

### Western blotting analysis

Protein samples were loaded in equal amounts onto SDS/PAGE gels and transferred onto polyvinylidene difluoride (PVDF) membranes. The membranes were blocked for 1 h with 5% skim milk. Subsequently, the PVDF membranes were treated with a primary antibody specific to the target protein and incubated overnight at 4 °C. The membranes were then washed three times with PBST and subsequently incubated with secondary antibody for 1 h at room temperature. The presence of the protein was detected using the ChemiDocTM Touch Imaging System (BIO-RAD) in conjunction with an enhanced chemiluminescence kit.

### Proteomic analysis

*Akk* and *Akk*-EVs samples (*n* = 3 for each group) were prepared as described previously and processed for proteomic analysis by Jingjie PTM BioLab (Hangzhou, China). Differentially enriched proteins were identified (*Akk*-EVs/*Akk* > 2 or < 0.5, *P* < 0.05). Functional annotation of proteins was performed in COG database. To investigate B2URF3-binding proteins in VSMCs, VSMCs were pretreated with osteogenic medium for 3 days and then harvested using IP lysis buffer containing PMSF and phosphate inhibitor cocktail. Lysed samples were incubated with or without the His-tagged B2URF3 recombinant protein at 4° overnight to form immunoprecipitated complexes. The immunoprecipitated complexes with His tag were incubated with anti-His magnetic beads at 4° for 4–6 h. Unbound samples were removed, and the magnetic beads were washed 3 times with lysis buffer and stored at −80 °C for proteomics analysis.

### Recombinant proteins, antibody targeting B2URF3 and Akk-EVs, plasmids and SiRNAs

Recombinant candidate proteins with His label and antibody targeting B2URF3 and *Akk*-EVs were commercially synthesised by Genscript ProBio Company (Nanjing, China). The small interfering RNA (siRNA) targeting TIMM50, ALDH1B1, RAB14 and PCBP2 were designed and synthesized by RiboBio™ (Guangzhou, China). The recombinant plasmids Flag-TIMM50 and Flag-ALDH1B1 were acquired from Genscript ProBio Company (Nanjing, China).

### Immunoprecipitation assay (IP)

Cells with or without plasmids Flag-TIMM50 and Flag-ALDH1B1 were incubated with 20 ug of His-tagged B2URF3 recombinant protein for 24 h and then were harvested with IP lysis buffer containing PMSF and phosphate inhibitor cocktail. After centrifugation at 12000 xg, 4 °C, for 10 min, a portion of the lysed sample was added to protein loading buffer as input, the remaining lysed sample was incubated with anti-Flag magnetic beads or anti-His magnetic beads in an inverted shaker at 4 °C overnight. The immunoprecipitated complexes were collected using a magnetic separator and washed three times with IP lysis buffer. The protein was eluted and denatured with 40 ul 1X SDS loading buffer, and then analyzed by western blotting.

### Competitive Enzyme-Linked immunosorbent assay

A competitive ELISA was performed to quantify the target antigen. High-binding 96-well plates were coated overnight at 4 °C with purified antigen (100 µL/well) in carbonate–bicarbonate buffer (0.05 M, pH 9.6), washed with PBST (PBS + 0.05% Tween-20), and blocked with 1x assay diluent for 1 h at room temperature. Serially diluted standards or pre-diluted samples were mixed (1:1) with a fixed concentration of primary antibody and incubated for 30–60 min at room temperature. The antigen–antibody mixtures (100 µL/well) were added to the coated plates and incubated for 1–2 h, followed by washing with PBST. A horseradish peroxidase -conjugated secondary antibody was added and incubated for 30–60 min, then plates were washed and developed using TMB substrate. The reaction was stopped with stop solution and absorbance was measured at 450 nm.

Percent inhibition was calculated relative to the no-competition control, and concentrations were interpolated from a four-parameter logistic (4-PL) standard curve.

### Protein extraction from human fecal samples

To determine the endogenous level of B2URF3 in the human gut, fecal samples were collected from healthy individuals. Approximately 1 g of stool was homogenized in 30 mL of ice-cold PBS containing a protease inhibitor cocktail. The suspension was mechanically dispersed using a 20-mL syringe plunger and transferred into a 50-mL tube. After centrifugation at 2,000 × g for 10 min at 4 °C, the supernatant was removed, and the pellet was resuspended in fresh PBS with protease inhibitors (final concentration: 1 g/mL). Samples were subjected to three cycles of freezing in liquid nitrogen (1 min), thawing at room temperature (20 min), and vortexing (5 s, maximum speed). The lysate was subsequently sonicated on ice (5 cycles, 20% amplitude, 30 s on/30 s off). Afterward, the mixture was centrifuged at 14,000 × g for 30 min at 4 °C, and the clarified supernatant was collected. Total protein concentration was quantified using a Bradford assay and used for subsequent Western blot analysis.

### Statistics analysis

All data were analyzed with GraphPad Prism 8.0 software. The Shapiro-Wilk Normality test was used to assess the distribution of the data. Data are expressed as the mean ± SD or median (25th to 75th quartiles) for continuous variables and n (%) for categorical variables, respectively. For continuous variables, Student’s t test or nonparametric Mann Whitney *U* test was used to make comparisons between two groups. One-way analysis of variance (ANOVA) with the Bonferroni post hoc test was used to make comparisons among three or more groups. For categorical variables, statistical significance was evaluated by χ2, or Fisher exact test. *P* value < 0.05 was considered statistically significant.

## Results

### Alleviation of vitamin D-induced vascular calcification by alternate-day fasting

**Fig. 1:**
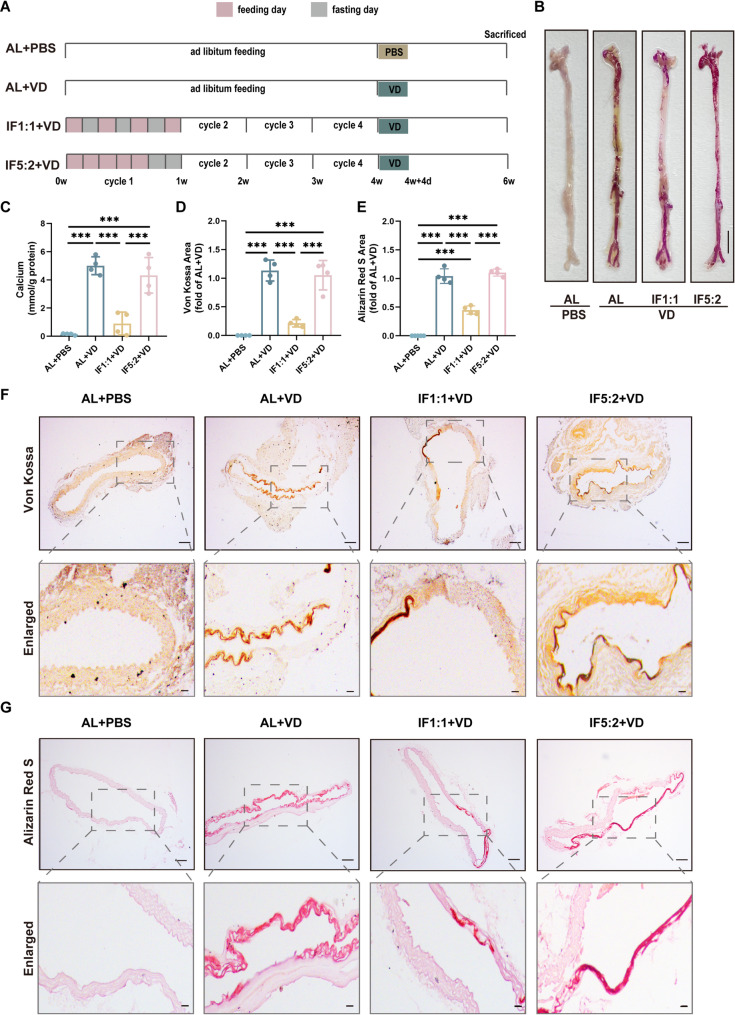
Alternate-day fasting attenuates vascular calcification. (**A**), Schematic of experimental design for assessing the effect of different fasting patterns on the VC model. (**B**), Representative image of the whole aorta stained with ARS. Scale bar = 5 mm. (**C**), Quantitative assessment of calcium content in the whole aorta. *n* = 4 for each group. (**D-E**), Quantification of Von Kossa and ARS staining area in each group relative to the AL + VD group. *n* = 4 for each group. (**F-G**) Von Kossa and ARS staining representative images of the aorta section. Scale bar = 100 μm (up) and 20 μm (bottom). Data are presented as the mean ± standard deviation (SD). Statistical significance was determined using one-way analysis of variance followed by Bonferroni *post hoc* test. ***P < 0.001

To investigate the effect of IF on VC, we generated VD-induced calcification mice and designed different dietary patterns to treat these mice (Fig. 1A). According to the body weight results, the weight of the mice in the IF5:2 + VD group was lower than that in the AL + VD group at the fourth week, and there was no significant difference in other groups (Fig.S1A). After 4 weeks, mice subjected to IF1:1 showed a significant decrease in blood glucose compared with AL-fed controls, whereas IF5:2 did not produce a significant change (Fig.S1B). These results demonstrate that the IF1:1 protocol effectively modulated systemic glucose metabolism, confirming the metabolic relevance of this fasting regimen. Compared with AL + VD, calcium salt deposition in the aorta was significantly reduced in IF1:1 + VD group, whereas no significant changes were observed in IF5:2 + VD group (Fig. 1B). Meanwhile, aortic calcium content showed obviously decreased in the IF1:1 + VD group relative to the AL + VD group (Fig. 1C). Similarly, Von Kossa and ARS of thoracic aortic sections from IF1:1 + VD mice and quantitative analysis showed that calcium salt deposition was significantly inhibited (Fig. 1D-F). The above results suggested that IF1:1 may alleviate VC.

### Gut microbiota mediates alternate-day fasting-mitigated vascular calcification

**Fig. 2:**
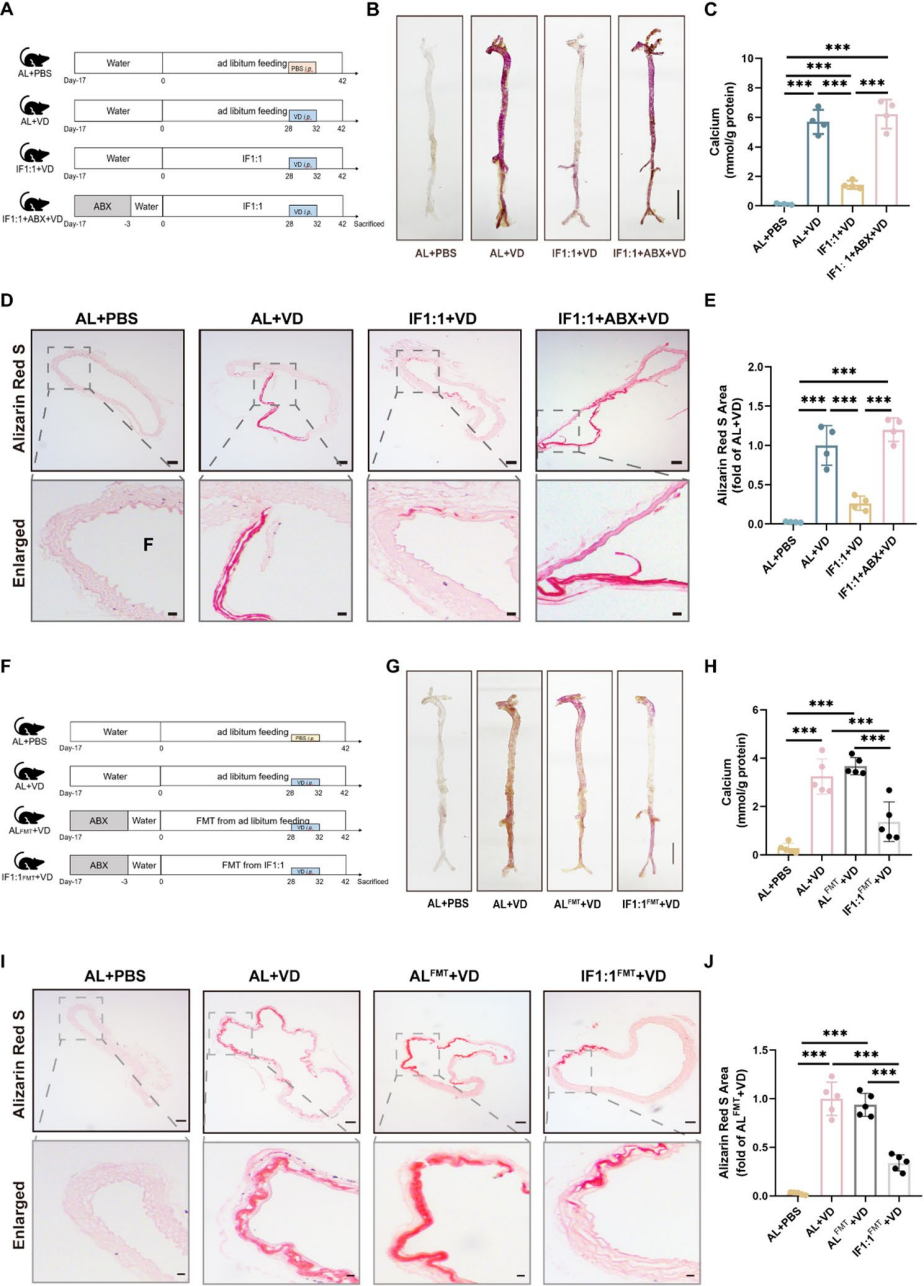
Gut microbiota mediates alternate-day fasting-mitigated vascular calcification. (**A**), Schematic of experimental design for assessing the antibiotic depletion assay. (**B**), Representative image of the whole aorta stained with ARS. Scale bar = 5 mm. (**C**), Quantitative assessment of calcium content in the whole aorta. (**D-E)**, Representative ARS-stained aortic sections and quantification of calcified area. Scale bar = 100 μm (left) and 20 μm (right). *n* = 4 for each group. (**F**), Schematic of experimental design for assessing the fecal microbiota transplantation (FMT) experiment. (**G**), Representative image of the whole aorta stained with ARS. Scale bar = 5 mm. (**H**), Quantitative assessment of calcium content in the whole aorta. *n* = 5 for each group. (**I-J**), Representative ARS-stained aortic sections and quantification of calcified area. Scale bar = 100 μm (left) and 20 μm (right). *n* = 5 for each group. Statistical significance was determined using one-way analysis of variance followed by Bonferroni *post hoc* test. Data are presented as the mean ± standard deviation (SD). ***P < 0.001

To elucidate the role of GM in mitigating VC by IF1:1, broad-spectrum antibiotics (ABX) were added to drinking water to clear GM in IF1:1-treated mice (Fig. 2A) [18]. Our previous research found that the ABX method can eliminate most of the intestinal flora in the body through 16 S rRNA sequencing [19]. Meanwhile, after four weeks of exposure to ABX, we collected feces for colony counts and observed that the number of fecal bacterial colonies in the IF1:1 + ABX + VD group was significantly reduced compared to the IF1:1 + VD group (Fig.S2A), indicating that most of the bacteria in the intestinal tract has been eliminated. Calcium salt deposition assays suggested that ABX abolished the inhibitory effect of IF1:1 on vascular calcification (Fig. 2B). In addition, the aortic calcium content of IF1:1 + ABX + VD mice was significantly higher than that of IF1:1 + VD group (Fig. 2C). ARS staining showed small ARS-stained areas in the thoracic aorta of IF1:1-treated mice, whereas the aorta of ABX-treated mice had larger areas of calcium deposition lesions stained with ARS (Fig. 2D, F). To further verify the important role of gut microbiota in IF1:1-mitigated VC, we transplanted feces from mice eating ad libitum to one group of mice before modeling vascular calcification (AL^FMT^+VD), and transplanted feces from mice fasted on alternate days to another group of mice (IF1:1^FMT^ + VD) (Fig. 2G). Additionally, ABX were used prior to transplantation to clear the mice of their native gut flora. Calcium salt deposition was significantly decreased in IF1:1^FMT^ + VD rather than AL^FMT^+VD (Fig. 2H). Furthermore, the results of calcium content detection and ARS were also consistent with the above results (Fig. 2I-J). Collectively, these results implicated that GM may play an essential role in the attenuation of VC by IF1:1.

### Alternate-day fasting alters the structure and composition of the gut microbiota and increases the abundance of *Akk* that attenuate vascular calcification

**Fig. 3:**
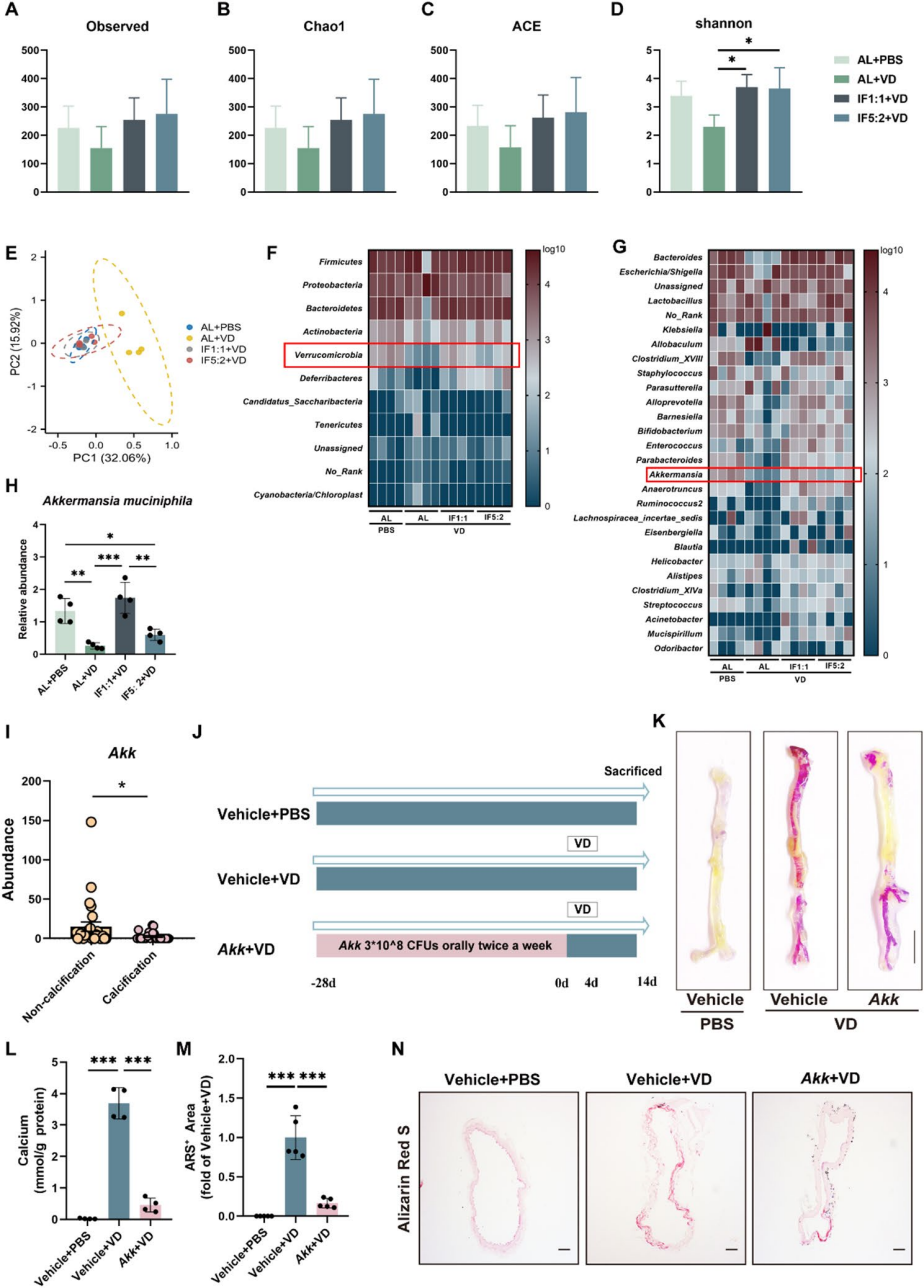
Alternate-day fasting alters the structure and composition of the gut microbiota and increases the abundance of *Akkermansia muciniphila* that resist vascular calcification. (**A to D**) Observed number of OTUs and estimated OTU richness (Chao1, ACE, and Shannon) in fecal microbiota of mice. *n* = 4 per group. (**E**) Principal coordinate analysis (PCoA) based on Bray-Curtis distances. *n* = 4 per group. (**F**) Composition of fecal microbiota at the phylum levels. (**G**), Composition of fecal microbiota at the genus levels. (**H**) qRT-PCR analysis of *Akk* abundance in fecal microbiota. *n* = 4 for each group. (**I**), Relative abundance of *Akk* in the fecal microbiota of patients with or without calcification. *n* = 27/27. (**J**), Schematic of experimental design for assessing the effect of *Akk* on the VC model. (**K**), Representative image of the whole aorta stained with ARS. Scale bar = 5 mm. (**L**), Quantitative assessment of calcium content in the whole aorta. *n* = 4 for each group. (**M-N**), Representative ARS-stained aortic sections and quantification of calcified area relative to the Vehicle + VD group. *n* = 5 for each group. Scale bar = 100 μm. Statistical significance was determined using one-way analysis of variance followed by Bonferroni *post hoc* test. Data are shown as mean ± SD. *P < 0.05, **P < 0.01, ***P < 0.001

To identify beneficial microbes that regulate the vasoprotective effects of IF1:1, we collected feces and assessed the microbial composition of these feces by 16 S rRNA high-throughput sequencing. The composition of the identified microbiota was analyzed in terms of biological classification. First, we evaluated the alpha diversity of the fecal microbiota in mice treated with different fasting regimens. The observed OTU numbers and estimated OTU abundances (Chao1, ACE, and Shannon) showed a downward trend after VD treatment compared with AL + PBS, but mice treated with IF1:1 and IF5:2 reversed this downward trend (Fig. 3A-D). Beta-diversity analysis utilizing the Bray–Curtis.

dissimilarity metric in principal coordinate analysis revealed distinct clustering of gut microbial profiles between different groups (Fig. 3E). At the phylum level, *Verrucomicrobia* was significantly higher in the IF1:1 + VD group, compared with the AL + VD groups (Fig. 3F). A consistent trend was also observed at the class, order, and family levels (Fig.S3A-C). At the genus level, *Akkermansia*, a well-studied genus of the V*errucomicrobia* phylum [20], was found to be significantly elevated in the IF1:1 + VD group (Fig. 3G). Furthermore, we found increased abundance of *Akkermansia muciniphila* (*Akk*) in the feces of mice in the IF1:1 + VD group versus the AL + VD group by quantitative real-time polymerase chain reaction (qRT-PCR) (Fig. 3H). By 16 S rRNA sequencing, we found that the abundance of *Akk* in the feces of mice that received IF1:1 fecal microbiota transplantation was higher than that of mice that received ad libitum diet (Fig.S4A-F).

To further explore the association between *Akk* abundance and VC, we collected feces samples from VC patients and controls. VC is an aging-related disease, so patients who develop VC are generally older. The patients who developed VC in the samples we collected were indeed significantly older than the control population. There were no differences in gender, BMI, systolic blood pressure (SBP), diastolic blood pressure (DBP), smoking, or diabetes between the two groups (Table.S1). 16 S rRNA sequencing analysis showed that the relative abundance of *Akk* was reduced in the feces of VC patients compared to control subjects (Fig. 3I). Given the evidence indicating that *Akk* in the gut microbiota may act as a protective factor against vascular calcification, we further investigated whether oral gavage of in vitro-cultured *Akk* could alleviate VC [21, 22] (Fig. 3J). qRT-PCR analysis showed that oral gavage transplantation of *Akk* significantly increased its colonization in the intestine of mice (Fig.S5). As evidenced by aorta calcium salt deposition (Fig. 3K), calcium content assay (Fig. 3L) and ARS (Fig. 3M-N), *Akk* treatment suppressed aortic calcium salt deposition and reduced calcium levels in vascular tissues of mice with vascular calcification. These results suggest that *Akk* may be associated with the vasoprotective effect of IF1:1, and supplementation with this probiotic may be a key mechanism for the anti-vascular calcification of IF1:1.

### EVs derived from *Akk* suppress both VSMC osteogenic transdifferentiation and subsequent vascular calcification

**Fig. 4:**
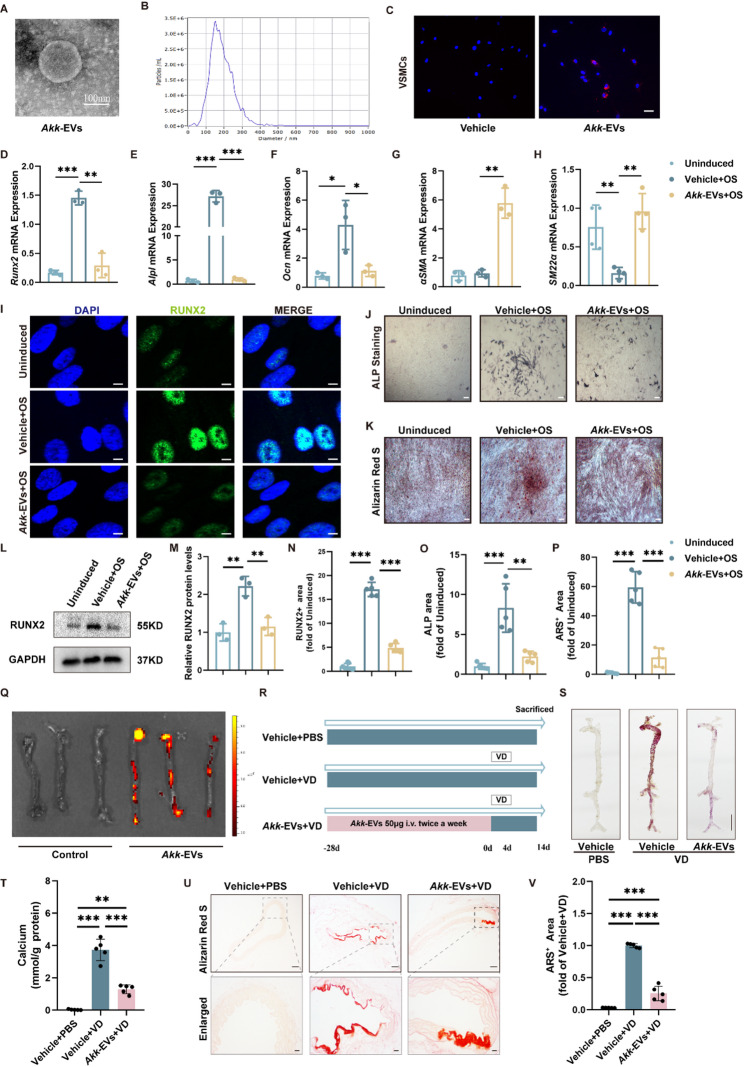
Inhibition of vascular smooth muscle cell osteogenic transdifferentiation by *Akk*-EVs. (**A**), Representative images of transmission electron microscopy in *Akk*-EVs. Scale bar = 100 nm. (**B**), The diameter distribution of Akk-EVs was measured by DLS analysis. (**C**), Representative images and quantification positive signals of A*kk*-EVs uptake by VSMCs after 12 h incubation. Nuclei stained by DAPI in blue and A*kk*-EVs stained by Dil in red.(**D-H**), mRNA level of *Runx2*,* Alpl*,* Ocn*, *SM22α* and *αSMA* in VSMCs treated with *Akk-*EVs. *n* = 3 or 4 for each group. (**I**), Representative images of RUNX2 immunofluorescence staining in VSMCs treated with *Akk-*EVs. Scale bar = 5 μm. (**J**), Representative images of ALP staining in VSMCs treated with *Akk-*EVs. Scale bar = 200 μm. (**K**), Representative images of ARS staining in VSMCs treated with *Akk-*EVs. Scale bar = 200 μm. **(L**), Protein levels of RUNX2 in VSMCs treated with *Akk-*EVs. (**M**), Quantification of RUNX2 protein level in each group. *n* = 3 for each group. (**N**), Quantification of RUNX2 positive area in each group. *n* = 5 for each group. (**O**), Quantification of ALP positive area in each group. *n* = 5 for each group. (**P**), Quantification of ARS positive area in each group. *n* = 5 for each group. (**Q**), Representative in vivo fluorescence image of *Akk-*EVs distribution in mice 24 h after *Akk-*EVs injection. (**R**), Schematic of experimental design for assessing the effect of *Akk*-EVs on the VC model. (**S**), Representative image of the whole aorta stained with ARS. Scale bar = 5 mm. (**T**), Quantitative assessment of calcium content in the whole aorta. *n* = 5 for each group. (**U-V**), Representative ARS-stained aortic sections and quantification of calcified area. Scale bar = 100 μm (up) and 20 μm (bottom). *n* = 5 for each group. Statistical significance was determined using one-way analysis of variance followed by Bonferroni *post hoc* test. Data are shown as mean ± SD. *P < 0.05, **P < 0.01, ***P < 0.001

Direct clinical use of *Akk* faces translational hurdles including strain-specific production and quality control, uncertainty about safety in vulnerable populations, and variable gut colonization. Consequently, the field has explored safer, more controllable alternatives such as microbe-derived products. EVs, acting as “nano-scale functional vehicles” secreted by the microbiota, transcend the spatiotemporal limitations of bacterial survival and directly mediate long-distance communication between the gut microbiota and host cells, serving as a critical entry point for investigating downstream mechanisms of the microbiota [23]. We isolated EVs from the culture medium (CM) in which *Akk* was cultured as described previously [15] and characterized the isolated EVs by using transmission electron microscopy and dynamic light scattering (DLS) analysis. As shown in Fig. 4A-B, *Akk*-EVs exhibit a spherical shape with a diameter of 152.6 ± 1.86 nm. To assess the effect of *Akk*-EVs on osteogenic differentiation of vascular smooth muscle cells (VSMCs), we first examined whether the red lipophilic dye DiI-labeled *Akk*-EVs could be internalized by VSMCs. As shown in Fig. 4C, the Dil-labeled *Akk*-EVs were presented at the perinuclear region of VSMCs, indicating the successful uptake of these EVs by VSMCs. qRT-PCR analysis showed that the mRNA levels of osteogenic markers (*Runx2*, Alkaline phosphatase (*Alpl*) and Osteocalcin (*Ocn*)) were significantly increased by osteogenic induction (OS) in VSMCs, however, this effect was reversed by *Akk*-EVs. Meanwhile, *Akk*-EVs enhanced the expression of contractile property markers (smooth muscle-22α (*SM22α*) and α-smooth muscle actin (*αSMA*)) (Fig. 4D-H). As shown in immunofluorescence staining (Fig. 4I, N), the green fluorescent signal labeled with RUNX2 was significantly reduced in osteogenic induced VSMCs treated with *Akk*-EVs compared to VSMCs after OS treatment alone. Furthermore, both alkaline phosphatase (ALP) staining and ARS staining observed a significant reduction of ALP positive area and calcium nodules in *Akk*-EVs treated relative to OS-treated VSMCs (Fig. 4J, K, O and P). Additionally, the protein expression level of RUNX2 was significantly reduced in the *Akk*-EVs + OS group (Fig. 4L and M).

Next, we performed in vivo experiments to directly investigate whether *Akk*-EVs could be internalized by arterial tissues and functionally modulate vascular calcification. DiR-labeled *Akk*-EVs were intravenously administered via the tail vein in mice, and their aortic localization was tracked. The results demonstrated that all *Akk*-EVs-treated mice exhibited distinct fluorescent signals in the artery compared to the vehicle control group (Fig. 4Q). The magnified histological images revealed the presence of *Akk*-EVs within the aortic tissue (Fig.S6). We further quantified the abundance of *Akk*-EVs across different fasting conditions. Immunofluorescence staining of aortic tissues revealed a significant reduction in EV signals in the AL + VD group compared with the AL + PBS group. Interestingly, the IF1:1 regimen restored Akk-EV levels, whereas the IF5:2 regimen did not produce a detectable increase in EV abundance (Fig.S7). We then examined whether tail vein injection of *Akk*-EVs could confer vascular benefits in VC mice (Fig. 4R). As evidenced by aorta calcium salt deposition (Fig. 4S), calcium content assay (Fig. 4T) and ARS (Fig. 4U-V), calcium deposition lesion areas were significantly decreased in *Akk*-EVs-treated mice compared to the Vehicle + VD group. Taken together, these results suggested that *Akk*-EVs protected against VC by suppressing osteogenic transdifferentiation of VSMCs.

### B2URF3 is enriched in *Akk*-EVs and May play an important role in alleviating vascular calcification

**Fig. 5:**
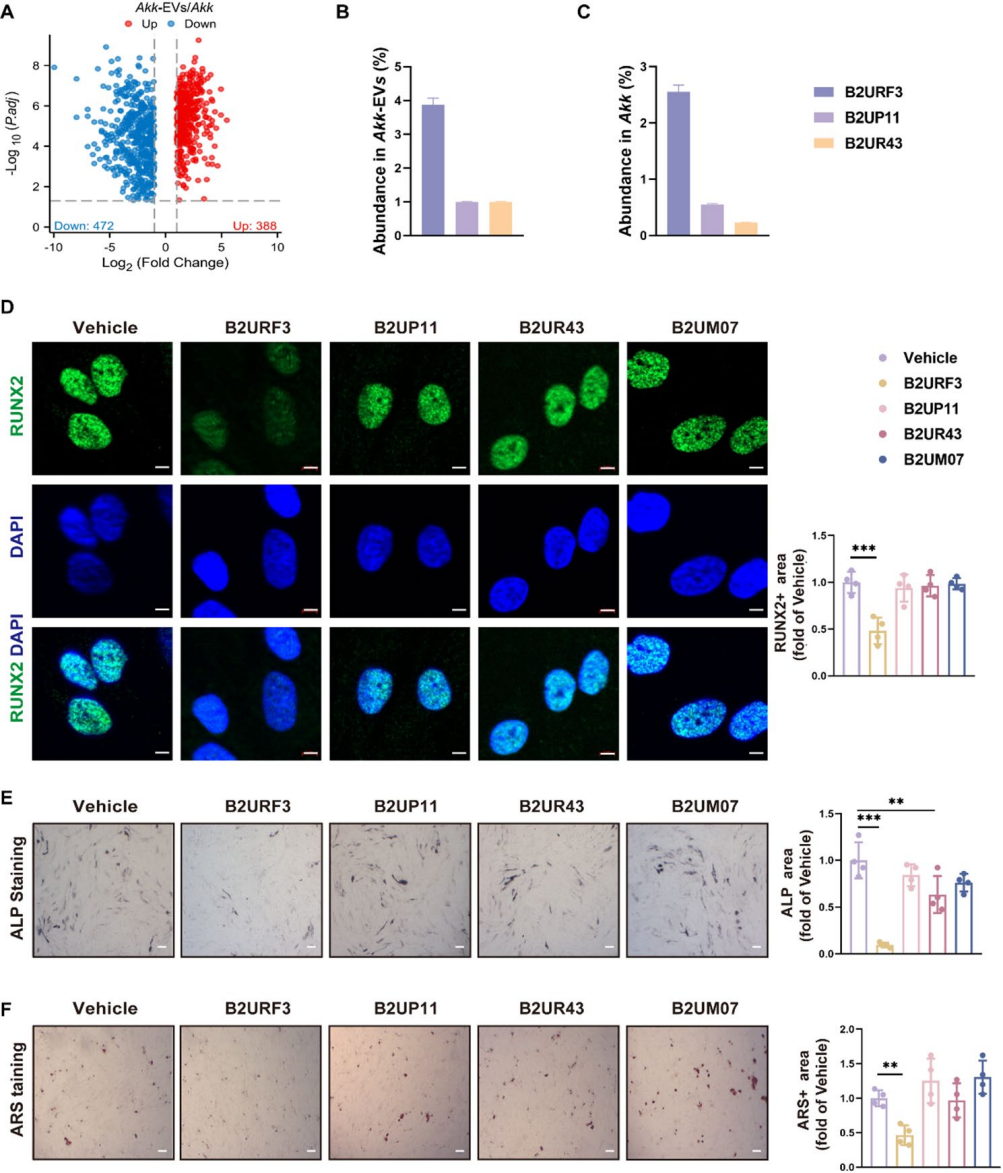
Highly enriched B2URF3 in *Akk*-EVs inhibits osteogenic transdifferentiation of VSMCs (**A**), Volcano plot of proteomics high-throughput sequencing (*Akk*-EVs group vs. *Akk* group). (**B**), Top three most abundant uncharacterized proteins in the *Akk*-EVs group. (**C**), Top three most abundant uncharacterized proteins in the *Akk* group. (**D**), Representative RUNX2 immunofluorescence staining and quantification of RUNX2-positive area relative to the Vehicle group. Scale bar = 5 μm. *n* = 4 for each group. (**E**), Representative images of ALP staining in VSMCs and quantification of ALP-positive area relative to the Vehicle group. Scale bar = 200 μm. *n* = 4 for each group. (**F**), Representative images of ARS staining in VSMCs and quantification of ARS-positive area relative to the Vehicle group. Scale bar = 200 μm. *n* = 4 for each group. Statistical significance was determined using one-way analysis of variance followed by Bonferroni *post hoc* test. Data are shown as mean ± SD. *P < 0.05, **P < 0.01, ***P < 0.001

In order to find the key effector molecules in *Akk*-EVs, we detected the protein composition of *Akk* and *Akk*-EVs through 4D non-standard quantitative proteomics high-throughput sequencing. Based on the sequencing, we found that 388 proteins were significantly upregulated in *Akk*-EVs compared to those in *Akk* (*Akk*-EVs/*Akk* > 2), while 472 proteins were markedly downregulated (*Akk*-EVs/*Akk* < 0.5) (Fig. 5A). Subsequently, we analyzed the abundance of upregulated proteins in *Akk*-EVs and *Akk.* By excluding those annotated with known functions in the COG (Clusters of Orthologous Groups) database, as well as highly conserved bacterial proteins and proteins localized to the bacterial periplasmic space, three highly abundant proteins with currently unknown functional annotations (B2URF3, B2UP11, and B2UR43) were ultimately selected (Fig. 5B-C). Besides, Previous studies have reported that B2UM07 (P9), that is secreted by *Akk*, may be a target for the treatment of metabolic diseases [24]. In order to explore the impact of the above four proteins on VSMCs calcification, we purified and synthesized recombinant proteins to intervene in VSMCs. The results showed that compared with the control group, B2URF3 reduced the protein level of RUNX2 in VSMCs, as evidenced by immunofluorescence staining of RUNX2 (Fig. 5D). Besides, B2URF3 treatment weakened decreased calcium deposits and the activity of ALP in VSMCs, as evidenced by ARS and ALP staining (Fig. 5E and F).

**Fig. 6:**
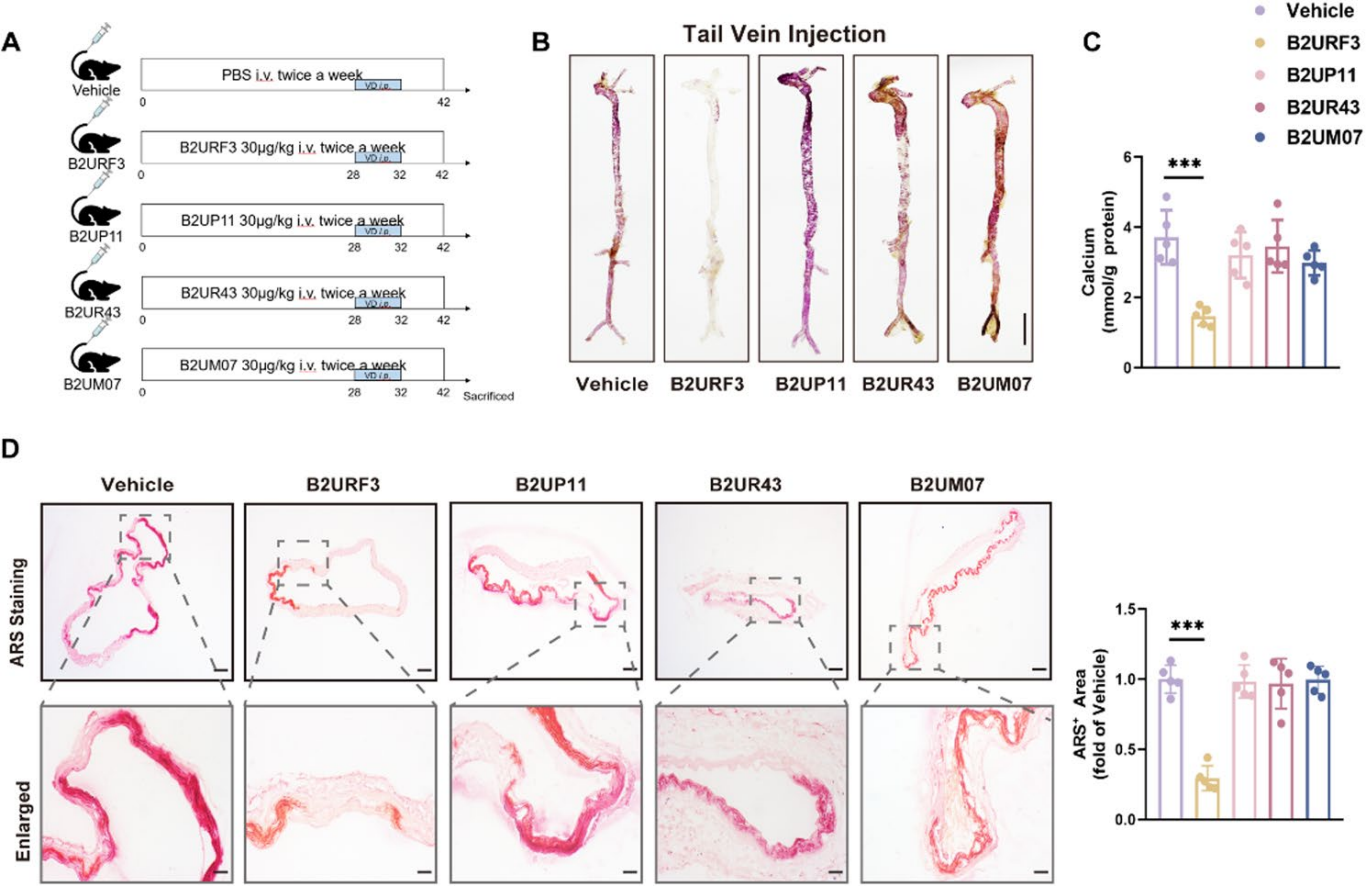
B2URF3 protects against vascular calcification in mice. (**A**), Schematic diagram of the experimental mechanism for tail-vein injection of uncharacterized proteins. (**B**), Representative image of the whole aorta stained with ARS. Scale bar = 5 mm. (**C**), Quantitative assessment of calcium content in the whole aorta. *n* = 5 for each group. (**D**), Representative ARS-stained aortic sections and quantification of calcified area. Scale bar = 100 μm (up) and 20 μm (bottom). *n* = 5 for each group. Statistical significance was determined using one-way analysis of variance followed by Bonferroni *post hoc* test. Data are shown as mean ± SD. *P < 0.05, **P < 0.01, ***P < 0.001

We intravenously administered B2URF3, B2UP11, B2UR43, B2UM07, or an equivalent volume of solvent via the tail vein to mice with induced vascular calcification. The injections were performed twice weekly for a total intervention period of four weeks. Whole aorta ARS results demonstrated that, compared to the vascular calcification model (Vehicle) group, mice treated with B2URF3 exhibited a significant reduction in aortic calcific deposition (Fig. 6A). Results from the calcium content assay and ARS of aortic sections showed that aortic calcium content in the B2URF3-treated group was significantly reduced compared to the Vehicle group. In contrast, B2UP11, B2UR43, and B2UM07 did not exhibit significant ameliorative effects on aortic calcification deposition or calcium content in mice with vascular calcification (Fig. 6B-D). Recombinant B2URF3 expression and purity were verified by SDS-PAGE and Western blot (Fig.S8). A single band at ~ 18–20 kDa was observed on SDS-PAGE, indicating high purity. Consistently, Western blot analysis using an anti-His antibody detected a single band at the same molecular weight, confirming the identity of the purified His-tagged B2URF3. To evaluate whether B2URF3 is present in the human microbiome, we extracted microbial proteins from stool samples of healthy individuals using a previously established extraction protocol [25]. Western blot analysis detected a clear B2URF3 band, demonstrating that B2URF3 is endogenously expressed within the human gut environment. This finding supports the translational relevance of our observations derived from murine and in vitro systems.

To investigate the correlation between B2URF3 with calcification, serum samples were collected from patients with coronary artery calcification (*n* = 15) and calcification-free controls (*n* = 16), and the levels of B2URF3 were measured. Analysis of clinical baseline characteristics showed that the mean age of the calcification group was higher than that of the control group, although the difference was not statistically significant. No significant differences were observed between the two groups in terms of gender, BMI, systolic blood pressure, diastolic blood pressure, smoking history, or prevalence of diabetes (Table.S2). ELISA test showed that the levels of B2URF3 in serum of VC patients were significantly lower than those in the control group (Fig.S10).

Because *Akk* naturally secretes EVs into the gut lumen, oral gavage delivery was included to reproduce this native exposure context. This strategy enabled us to evaluate whether enteral supplementation of EVs could elicit protective effects against VC, comparable to those achieved through systemic delivery. To assess whether oral *Akk*-EVs could be detected in the mouse aorta. Immunofluorescence staining for polyclonal antibody targeting *Akk*-EVs prepared in a previous study [15] revealed that fluorescent signals were able to be detected in the aorta of vehicle- and *Akk*-EVs-treated mice, and higher signals were observed in *Akk*-EVs-treated mice relative to vehicle-treated mice (Fig. S11). This suggests that *Akk*-EVs can be delivered to the host aorta under physiological conditions, while exogenous oral supplementation with *Akk*-EVs treatment further increases the abundance in the aorta. In addition, we evaluated the effects of orally administered *Akk*-EVs, B2URF3, B2UP11, B2UR43, B2UM07, or an equivalent volume of solvent on VC in mice (Fig.S12 A). Analysis of whole aorta ARS revealed that, compared with the VC model (Vehicle) group, oral administration of *Akk*-EVs significantly reduced calcium salt deposition in the aortas of mice with VC. Further investigation demonstrated that among the candidate proteins, B2URF3 significantly decreased aortic calcification deposition, while B2UR43 exhibited a moderate reduction in calcium deposition. In contrast, B2UP11 and B2UM07 did not show significant ameliorative effects on calcification (Fig.S12 B). Moreover, calcium content assays indicated that the aortic calcium levels were significantly lower in the *Akk*-EVs, B2URF3, and B2UR43 groups compared to the Vehicle group, whereas no significant changes were observed in the B2UP11 and B2UM07 groups (Fig.S12 C-E). Taken together, these results suggest that B2URF3 may play an important role in *Akk*-EVs alleviating VC.

### ALDH1B1 binds to B2URF3 and May play an important role in B2URF3 inhibiting VSMC osteogenesis

**Fig. 7:**
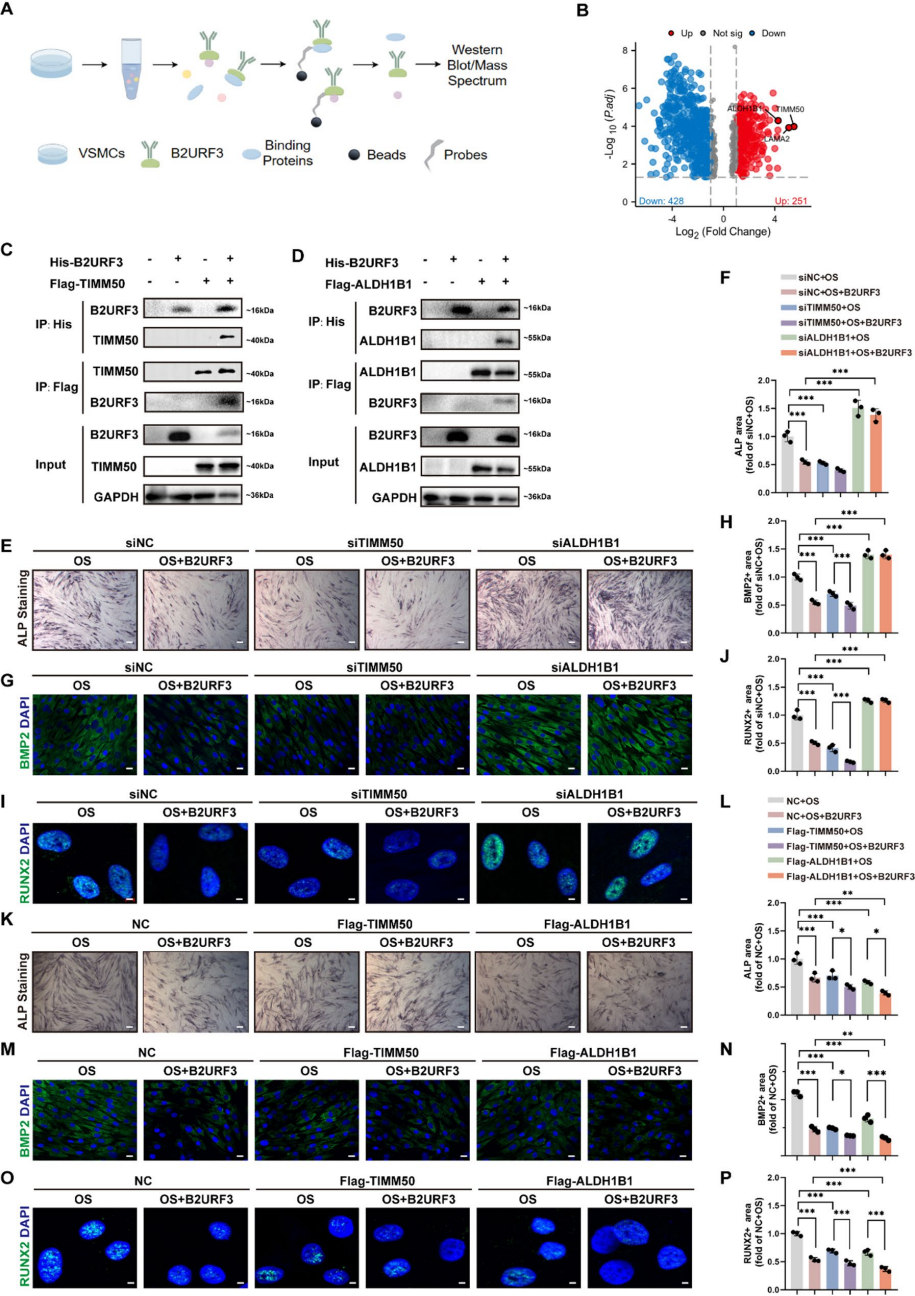
ALDH1B1 binds to B2URF3 and may play an important role in B2URF3 inhibiting VSMCs osteogenesis. (**A**), Schemes of immunoprecipitation-mass spectrometry assay. (**B**), Volcano plot of proteomics high-throughput sequencing. (**C**), IP assay showing the interaction between His-B2URF3 and Flag-TIMM50. (**D**), IP assay showing the interaction between His-B2URF3 and Flag-ALDH1B1. (**E-F**), Representative images of ALP staining in VSMCs and quantification of positive area in VSMCs transfected with siRNA. Scale bar = 200 μm. *n* = 3 for each group. (**G-H**), Representative BMP2 immunofluorescence staining and quantification of positive area in VSMCs transfected with siRNA. Scale bar = 5 μm. *n* = 3 for each group. (**I-J**), Representative RUNX2 immunofluorescence staining and quantification of positive area in VSMCs transfected with siRNA. Scale bar = 5 μm. *n* = 3 for each group. (**K-L**), Representative images of ALP staining in VSMCs and quantification of positive area in VSMCs transfected with overexpression plasmids. Scale bar = 200 μm. *n* = 3 for each group. (**M-N**), Representative BMP2 immunofluorescence staining and quantification of positive area in VSMCs transfected with overexpression plasmids. Scale bar = 5 μm. *n* = 3 for each group. (**O-P**), Representative RUNX2 immunofluorescence staining and quantification of positive area in VSMCs transfected with overexpression plasmids. Scale bar = 5 μm. *n* = 3 for each group. Statistical significance was determined using one-way analysis of variance followed by Bonferroni *post hoc* test. Data are shown as mean ± SD. *P < 0.05, **P < 0.01, ***P < 0.001

Subsequently, we investigated whether B2URF3 binds to proteins to exert its efficacy by immunoprecipitation-mass spectrometry (IP-MS) analysis (Fig. 7A). Silver staining and mass spectrometry suggested that multiple proteins bound to B2URF3 (Fig.S13 and Fig. 7B). IP-MS results showed that compared with the control group, the proteins that bound most to B2URF3 protein were Translocase of inner mitochondrial membrane 50 (TIMM50), Laminin Subunit Alpha 2 (LAMA2), ALDH1B1. Considering that previous studies have found that LAMA2 may be involved in inhibiting osteogenic transformation [26], we hope to explore more innovative targets, so this study did not include it in subsequent studies. To assess the direct binding of B2URF3 to TIMM50 and ALDH1B1, we transfected Chinese Hamster Ovary Cell (CHO) with FLAG-TIMM50 and FLAG-ALDH1B1 expression plasmids respectively before B2URF3 treatment, and then collected the cell lysate for immunoprecipitation, which was followed by western blotting. We observed that both TIMM50 and ALDH1B1 could bind to B2URF3 (Fig. 7C-D). To investigate whether TIMM50 or ALDH1B1 is the key molecule mediating the effect of B2URF3 on osteogenesis, we transfected VSMCs with small interfering RNA (siRNA) to knockdown TIMM50 or ALDH1B1 and then induced these VSMCs to undergo osteogenic differentiation in the presence of B2URF3. In order to screen out the siRNA sequence with the best knockdown efficiency, we used qRT-PCR to detect the effects of different sequences. The results showed that siTIMM50-2 and siALDH1B1-2 had better knockdown effects than the other sequences, so we chose this sequence in subsequent experiments(Fig.S14 A-B). As ALP staining results showed, B2URF3 significantly inhibited VSMC osteogenesis, and the inhibitory effect of B2URF3 was abolished after knockdown of ALDH1B1, while knockdown of TIMM50 had no effect (Fig. 7E-F). SiRNA knockdown of ALDH1B1 significantly increased B2URF3-induced reduction in RUNX2 and Bone Morphogenetic Protein 2 (BMP2) protein levels, which was confirmed by immunofluorescence staining of RUNX2 and BMP2 (Fig. 7G-J). Moreover, we transfected VSMCs with FLAG-TIMM50 and FLAG-ALDH1B1 expression plasmids to overexpress TIMM50 and ALDH1B1. ALP staining results showed that the inhibitory effect of B2URF3 on osteogenesis was further enhanced after overexpression of ALDH1B1 (Fig. 7K-L). Immunofluorescence staining results showed that overexpression of ALDH1B1 enhanced the inhibition of BMP2 and RUNX2 protein levels by B2URF3 (Fig. 7M-P). Furthermore, we conducted molecular docking analysis to determine whether B2URF3 directly interacts with ALDH1B1. The results of molecular docking demonstrated the existence of multiple binding sites between B2URF3 and ALDH1B1(Fig.S15). Taken together, these results suggest that ALDH1B1 may bind to B2URF3 and thus mediate the inhibitory effect of B2URF3 on VSMCs osteoblastogenesis.

## Discussion

In the current study, we confirmed the beneficial role of IF1:1, but not IF5:2, in protecting against VC. Fecal microbiota transplantation experiment confirmed that the intestinal flora is involved in the vascular protective effect of IF1:1. In addition, IF1:1 significantly increased the abundance of *Akk* in feces, and the abundance of *Akk* in VC patients was reduced compared with the control group. Direct supplementation of this bacterium and its derived extracellular vesicles reduced calcium deposition in vivo and in vitro. Notably, *Akk* and its derived extracellular vesicles could generate a novel protein, B2URF3, which directly binds to ALDH1B1, ultimately protecting against VC. These findings may create new ideas for the prevention and treatment of VC.

The amount of food consumed and the time window in which it is consumed are key determinants of cardiovascular health. Stekovic et al. carried out a clinical study in healthy non-obese individuals found positive alterations in cardiovascular disease risk factors and in fat mass after only 4 weeks of IF1:1 [27]. Heilbronn et al. confirmed that time-restricted intermittent fasting has certain benefits in improving postprandial glucose metabolism and insulin sensitivity in adults at high risk for Type 2 diabetes mellitus [28]. Previous studies have shown that different fasting patterns lead to some degree of weight loss, and our results are consistent with that, but not statistically different. We think this may be due to the short duration of the intervention. Although some studies have shown that IF5:2 and IF1:1 have similar health-improving effects, some literature also found differences between the two regimens. Davies et al. found that the IF5:2 did not increase adult hippocampal neurogenesis or enhance memory in mice [29]. But Serger et al. showed that IF1:1 promotes axonal regeneration and repair [30] and Pan et al. confirmed IF1:1 ameliorated age-related cognitive decline [31]. This may be due to the fact that compare to the IF1:1 regimen, IF5:2-fed mice received less frequent dietary restrictions, with lengthened ad libitum feeding intervals in between, were insufficient to achieve the effect of reducing calcium deposition, or that the 4-week period was an insufficient length of time for the IF5:2 regimen to protect against VC. Taken together, these results suggest that IF1:1 plays an important role in improving VC.

Short-term dietary changes have a direct impact on microbiota diversity, while long-term dietary habits have a significant impact on microbiota composition [32]. Results from animal studies indicate that IF can have a positive impact on the microbiota by promoting diversity and modulating the abundance of specific bacterial species. These effects may be particularly helpful in minimizing the effects of a high-fat diet on the microbiota and reducing the abundance of obesogenic bacteria [33, 34]. In an Alzheimer’s disease-like mouse model, IF led to an increase in protective bacteria belonging to the genus *Lactobacillus* [31]. In this study, we found that after eliminating the endogenous intestinal flora of mice using antibiotics, the inhibitory effect of IF1:1 on VC was reversed. we found that the gut-microbiota-vasculature axis is involved in the protective effect of IF1:1 against VC by fecal microbiota transplantation experiment. When feces from IF1:1 mice were transplanted into mice that had been pre-treated with antibiotics, calcium deposits in the aorta were significantly reduced compared to the group received feces from mice that were fed ad libitum. These results suggest that GM contains protective factors in the vascular protective effects of IF1:1. Changes in GM after IF1:1 was detected by 16 s rRNA sequencing. Previous literature has reported that *Akk* abundance changes with different dietary patterns. Mousavi et al. found that fasting during Ramadan as an IF regimen promotes increased abundance of *Akk* [12]. Su et al. observed that *Akk* levels were strongly upregulated in the fasting volunteers [35]. Consistently, in our study, we found that *Akk* abundance was higher in IF1:1 mouse, which was also verified by qRT-PCR, possibly as a consequence of the competitive advantage this organism obtains in the absence of food as it can degrade intestinal mucins. Since its discovery in 2004, *Akk* has been recognized as a “next-generation probiotic” due to its beneficial regulatory effects in metabolic and immune-related diseases [36]. Recent evidence has expanded this concept by suggesting that *Akk* may influence distant organs beyond the gut, including the cardiovascular system. Several preclinical studies have demonstrated that *Akk* supplementation attenuates atherosclerotic lesion formation, improves endothelial function, and modulates systemic inflammation by restoring intestinal barrier integrity and reducing lipopolysaccharide (LPS) translocation [37–40]. A recent study by Yan et al. demonstrated that intragastric administration *Akk* ameliorated VC in animal models [41], supporting this emerging gut–vascular axis. Moreover, emerging evidence indicates that beyond the *Akk* bacteria themselves, its microbial derivatives—including metabolites and EVs—may provide comparable or even superior biological activity with improved translational feasibility. Consistent with these findings, our clinical data revealed a decreasing trend of *Akk* abundance in the feces of patients with VC, and fecal microbiota transplantation using an *Akk*-enriched community significantly reduced aortic calcium deposition in mice. Together, these results imply that strategies aimed at restoring or increasing Akk abundance may hold therapeutic potential for mitigating VC. Furthermore, it is worth noting that in addition to *Akk*, many other bacteria may also be involved in the regulation of VC, and more functional bacteria should be disclosed in future work.

Interestingly, whole-aorta imaging revealed a heterogeneous response to the intervention, with a more pronounced reduction in calcification within the midportion (descending thoracic aorta) compared to the proximal and distal segments. This may be related to the hemodynamic differences in different segments of the aorta and the biological heterogeneity of the vessel wall itself. Wall shear force is the parallel frictional force generated by blood flow against the vessel wall. Studies have shown that the ascending aorta and aortic arch, due to their tortuous geometry and the openings of major branches, exhibit complex blood flow patterns, easily generating low or oscillating wall shear forces [42]. Such hemodynamic environments can promote local inflammation, increased permeability and phenotypic switching of VSMCs toward an osteogenic program, all of which favour focal calcification. In contrast, the blood flow in the thoracic segment of the descending aorta is relatively stable, with higher and more consistent wall shear forces, which maintain an anti-inflammatory and anti-calcification endothelial phenotype and may facilitate a more detectable therapeutic response.

The mechanisms through which GM exerts protective effects on VC are still being investigated. Our research found that *Akk*-EVs can be taken up by VSMCs, thereby inhibiting osteogenic formation. These findings demonstrate that *Akk*-EVs communicate with the host’s distal organs. An important mechanistic question is how *Akk*-EVs reach VSMCs in vivo. Recent mechanistic and in vivo studies indicate that microbiota-derived EVs can traverse the intestinal barrier and access systemic circulation by multiple non-exclusive routes. EVs may be internalized by intestinal epithelial cells and undergo transcytosis, translocated via M cells overlying Peyer’s patches, or be captured and transported by mucosal immune cells into the lamina propria before entry into lymphatic or blood vessels; additionally, increased epithelial permeability (e.g., during inflammation) can facilitate paracellular leak of nanoscale vesicles [43, 44]. These processes have been demonstrated in both mechanistic cell models and animal studies [45, 46], and *Akk*-EVs in particular have been shown to interact with and modulate intestinal epithelial barrier function, supporting their capacity to be taken up at the gut interface. Taken together, these data provide a plausible physiological pathway by which *Akk*-EVs could translocate from the gut lumen into the bloodstream and subsequently be delivered to vascular walls to exert direct effects on VSMCs; nevertheless, direct in vivo tracing in our model would strengthen causal attribution and is proposed as future work. Moreover, as natural nanocarriers, *Akk*-EVs can effectively encapsulate and deliver drugs or bioactive molecules in a targeted manner, enhancing delivery efficiency while reducing cytotoxicity due to their high biocompatibility. However, research and application of GM-derived EVs face challenges. The complexity and diversity of bacterial communities, coupled with environmental sensitivity, may alter the composition and function of EVs. Thus, further optimization of isolation and purification protocols is essential to ensure consistency and reproducibility, facilitating their reliable biomedical application.

Given the inherent limitations of *Akk*-EVs, we hypothesized that they may be enriched with specific components that inhibit vascular calcification. To further elucidate the composition of *Akk*-EVs, we employed 4D label-free quantitative proteomics combined with high-throughput sequencing to analyze their protein profiles. Through in vivo and in vitro experiments, we identified and validated B2URF3 as a key protein in *Akk*-EVs that inhibits osteogenic differentiation. To our knowledge, this protein (according to UniProt) is annotated in *Akk* strain as gene ‘*Amuc_1213*’. However, the gene sequence of *Amuc_1213* is currently unclear, and there are no reported studies on it. Further research is needed in the future. Our results demonstrated that both intravenous injection and oral administration of B2URF3 effectively attenuated VC. The dual-route design allowed mechanistic differentiation between gut-mediated and circulation-mediated biological effects of EVs. Oral delivery evaluated whether EVs could engage the endogenous gut–vascular axis, while intravenous delivery to examine systemic bioavailability and direct vascular activity. Collectively, these data indicate that the potential of B2URF3 as an emerging biological agent for vascular anti-calcification intervention. In addition to experimental validation, our clinical observations further support the translational relevance of B2URF3. We detected B2URF3 in human fecal microbiota extracts, demonstrating that this protein is physiologically present within the human gut microbial environment rather than an artifact of experimental culture. Notably, serum B2URF3 levels were significantly reduced in individuals with coronary calcification compared with non-calcified controls, suggesting that diminished production, release, or systemic transport of microbiota-derived B2URF3 may be associated with calcification severity. Given that *Akk* abundance was also decreased in calcified subjects, these findings align with the proposed gut–vascular axis model and raise the possibility that loss of B2URF3-mediated signaling contributes to vascular calcification progression. Together, these data indicate that B2URF3 may serve not only as a surrogate marker reflecting microbial–host communication but also as a candidate protective effector with diagnostic and therapeutic potential in VC.

In order to further explore the potential action pathway of B2URF3 in vascular protection, we identify ALDH1B1 as a potential interacting partner of B2URF3, supported by IP-MS, co-immunoprecipitation, knockdown, overexpression, and in silico docking analyses. These observations suggest a mechanistic link in which ALDH1B1 may participate in mediating the anti-osteogenic effects of B2URF3 in VSMCs. ALDH1B1, initially characterized in 1991, is located in the 9.p13.1 chromosome region and lacks introns [47]. Although ALDH1B1 is predominantly expressed in the liver, pancreas, and kidney, previous [48, 49], studies have demonstrated that it is also detectable in arterial and atherosclerotic plaque tissues, where its expression is downregulated during the progression of atherosclerosis [50]. These findings indicate that ALDH1B1 may possess potential biological relevance within the pathological vascular microenvironment. ALDH1B1 is known to participate in the oxidation of lipid peroxidation products such as 4-hydroxynonenal (4-HNE) and malondialdehyde (MDA). Lipid peroxidation has been implicated in a variety of pathological processes, including atherogenesis [51]. MDA can be oxidized by ALDH followed by decarboxylation to generate CO2 and acetate [52]. In our preliminary observations, acetate appeared to be associated with reduced calcium salt deposition and osteogenic differentiation of VSMCs, suggesting a potential protective effect on vascular calcification [19]. Based on these findings, it is possible that the interaction between B2URF3 and ALDH1B1 could be involved in pathways related to oxidative stress and glutathione metabolism. However, although our data strongly support a functional association between B2URF3 and ALDH1B1, they do not yet establish a definitive causal hierarchy or exclude the involvement of additional intermediary pathways. Future studies employing genetic loss- and gain-of-function models in vivo and structural binding validation will be necessary to determine whether ALDH1B1 functions as the primary and direct mediator of B2URF3 activity during VC.

There are still some limitations in our study. First, there are many models used to simulate vascular calcification [53], in this study we only used the VD-induced VC model because this model is characterized by a short cycle and a high success rate. However, due to the complexity of the pathogenesis of vascular calcification, whether these results are also applicable to other models remains unknown and needs further exploration. Although we focused on the anti-calcification effect of *Akk* in this study, due to the lack of antibiotics specifically targeting *Akk*, we cannot rule out the existence of other bacteria that may play a protective role. EV-related studies are based on experiments using EVs isolated from in vitro bacterial cultures. However, the composition of EVs is closely related to their growth environment [54]. Whether the physiological properties of EVs secreted in vitro are the same as those of EVs secreted in vivo require further study. Another limitation of our study is that we did not perform a “dose-response” experiment to more carefully evaluate the effects of *Akk*-EVs and B2URF3 on vascular phenotypes in the normal physiology and pathology of vascular calcification. Currently, there is no evidence of physiological concentrations of *Akk*-EVs and B2URF3 in vascular tissue. Future studies need to accurately determine the physiological concentrations of *Akk*-EVs and B2URF3 and study whether there is a dose-dependent response in mice treated with *Akk*-EVs and B2URF3, which will help to more deeply interpret the functional role of *Akk*-EVs in VC and to develop strategies utilizing *Akk*-EVs and B2URF3 for the treatment of VC. This study incorporated IF, oral gavage, and intravenous EV delivery as part of a sequential, hypothesis-driven workflow. While this stepwise design enabled us to dissect the relative contributions of host metabolic state, microbial colonization, and vesicle-mediated signaling, it does not fully represent a single coherent physiological pathway. Future work will focus on streamlined experimental systems—particularly gut-anchored EV transport models—to better model the natural gut-vascular axis.

## Conclusion

In summary, we provide new evidence that IF protects against VC by regulating intestinal bacterial *Akk*. Shaping the GM through dietary intervention may be an attractive, effective and non-invasive strategy to prevent VC. By revealing the anti-calcification effects of B2URF3, a novel protein derived from *Akk*, we further broadened the potential mechanism by which *Akk* exerts its protective effects. B2URF3 may serve as a potentially effective treatment for VC if exerting protective effects on VC in future clinical practice.

## Supplementary Information

Supplementary material 1.

## Data Availability

No datasets were generated or analysed during the current study.
