# Supplementary material for: A novel protein B2URF3 from Akkermansia muciniphila increased by intermittent fasting alleviates vascular calcification
Source: J Nanobiotechnology. 2026 Jan 7;24:21. doi: 10.1186/s12951-025-03948-0 (PMC12781770; doi:10.1186/s12951-025-03948-0)
Supplement: Supplementary file 1 — Supplementary material 1. [file 12951_2025_3948_MOESM1_ESM.docx]

Supplement materials.


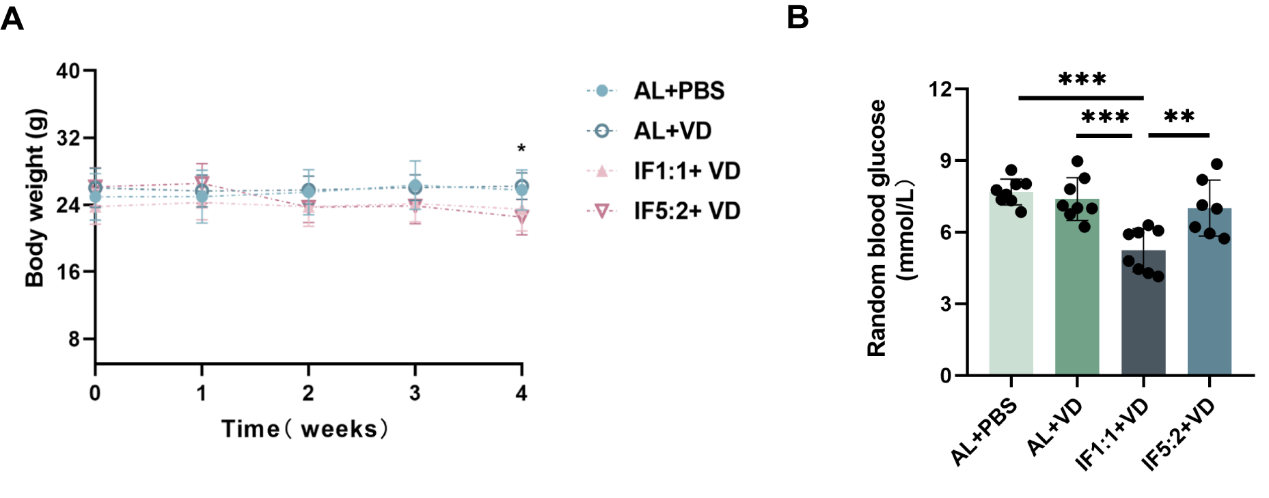


Fig.S1 Body weight (A) and blood glucose (B) changes during intermittent fasting intervention. Statistical significance was determined using one-way analysis of variance followed by Bonferroni post hoc test. **P* < 0.05; ***P* < 0.01; ****P* < 0.001.


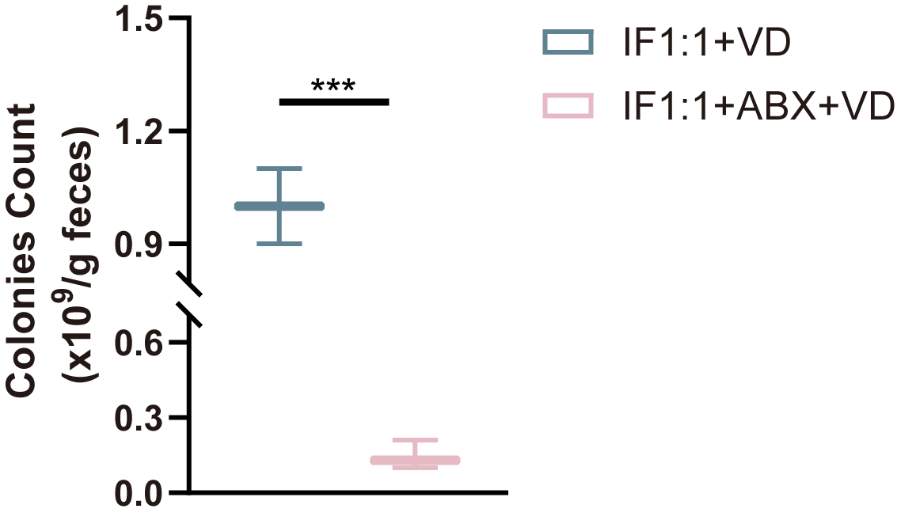


Fig.S2 Eliminating the gut microbiota of mice with a cocktail of antibiotics. n = 8 for each group. Data are presented as mean ± SD. Student’s t test was used to make comparisons between two groups. ****P* < 0.001.


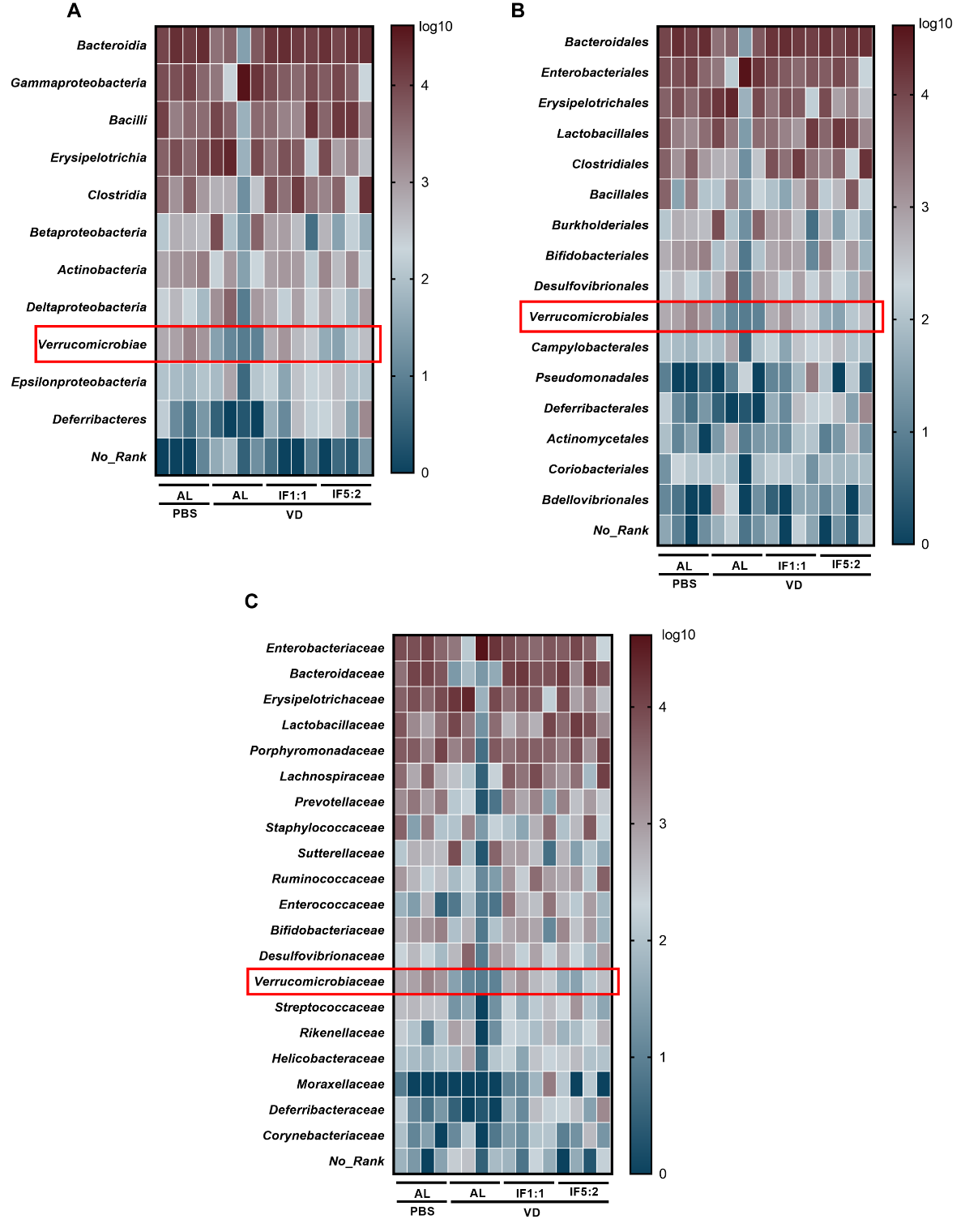


Fig.S3 Alternate-day fasting reshapes the gut microbiota and increases *Akk* abundance. (A), Class level. (B), Order level. (C), Family level.


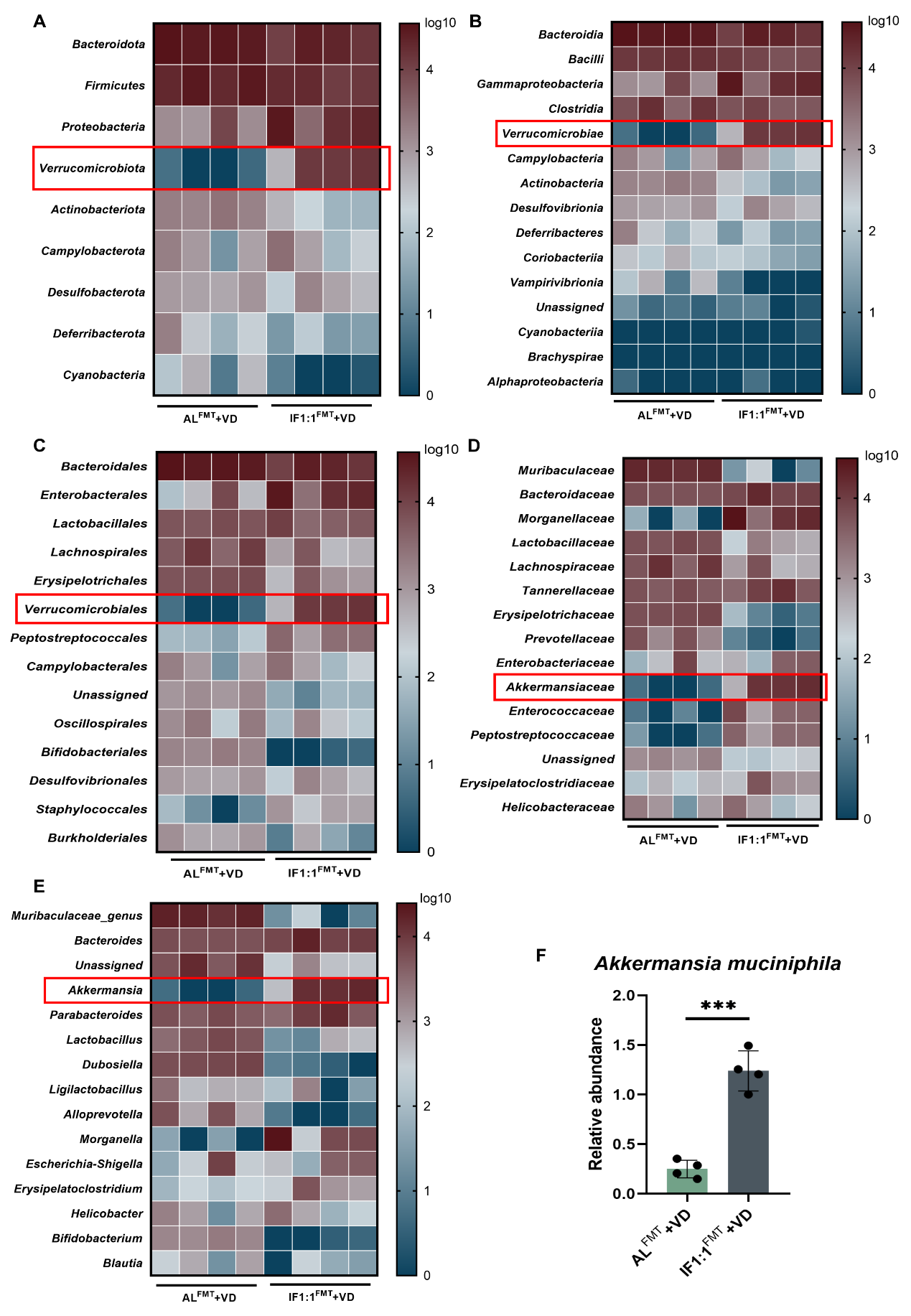


Fig.S4 Fecal Transplantation after Alternate-Day Fasting boosts *Akk* abundance in mice versus Ad Libitum Diet. (A), Phylum level. (B), Class level. (C), Order level. (D), Family level. (E), Genus level. (F), qRT-PCR analysis of *Akk* abundance in fecal microbiota. n = 4 for each group. Student’s t test was used to make comparisons between two groups. ****P* < 0.001.


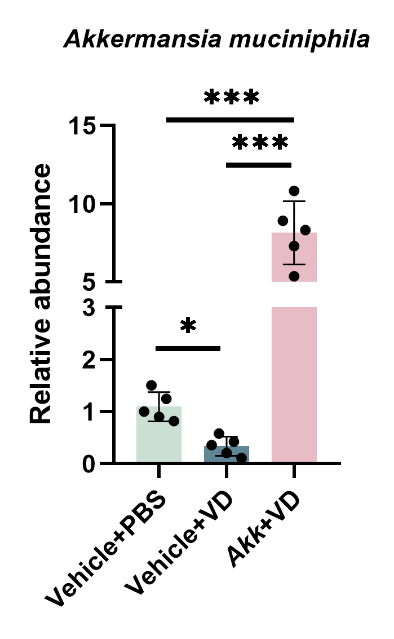


Fig.S5 qPCR validation of colonization in mouse feces following oral gavage.

Statistical significance was determined using one-way analysis of variance followed by Bonferroni post hoc test. Data are presented as mean ± SD. **P* < 0.05, ****P* < 0.001.


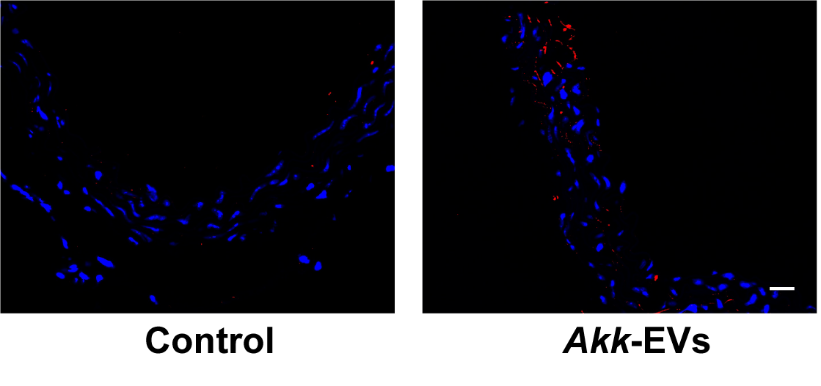


Fig.S6 Representative images of the *Akk*-EVs antibody (Ab)-stained aortic section after *AKK*-EVs intravenous injection. Scale bar = 20 μm.


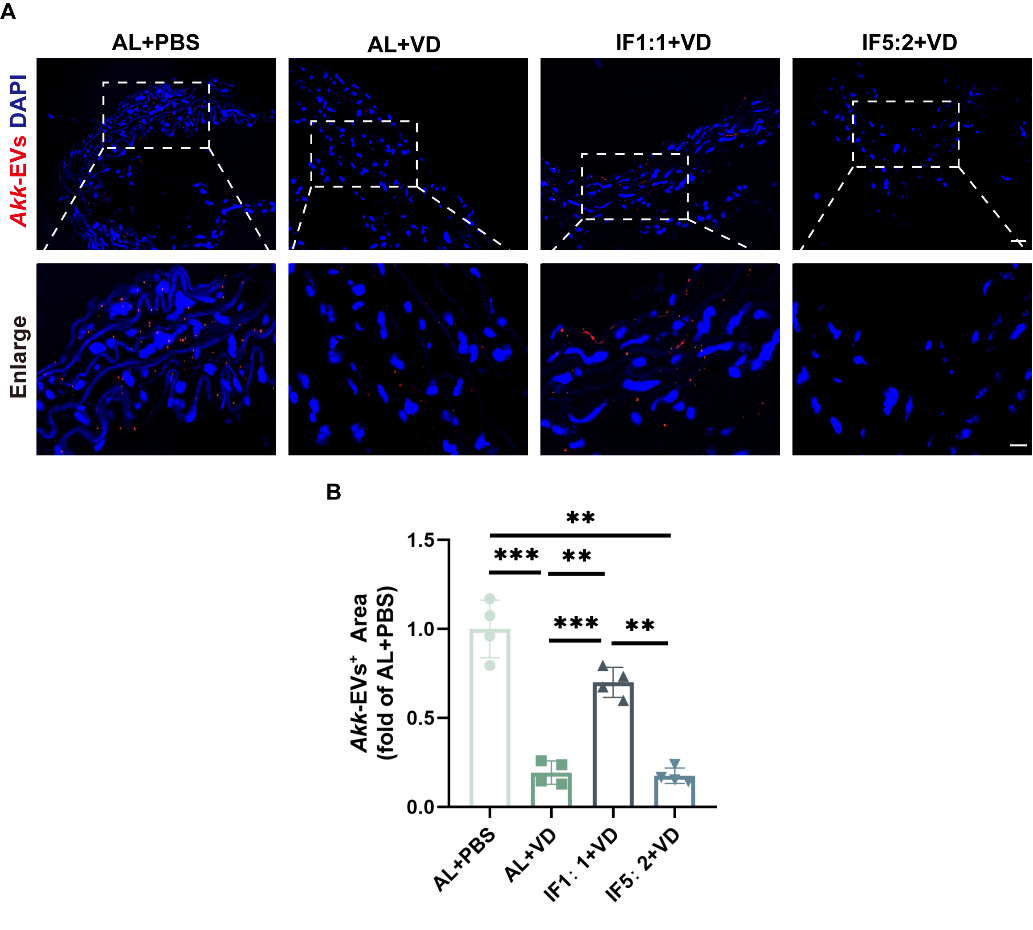


Fig.S7 Levels of *Akk* -EVs in aortic tissue under different fasting regimens.

(A) Representative immunofluorescence images showing *Akk*-EV localization in mouse aortas. Scale bars = 20 μm (up) and 10 μm (bottom).

(B) Quantification of relative *Akk*-EV fluorescence intensity in aortic tissue. Statistical significance was determined using one-way analysis of variance followed by Bonferroni post hoc test. Data are presented as mean ± SD. ***P* < 0.01, ****P* < 0.001.


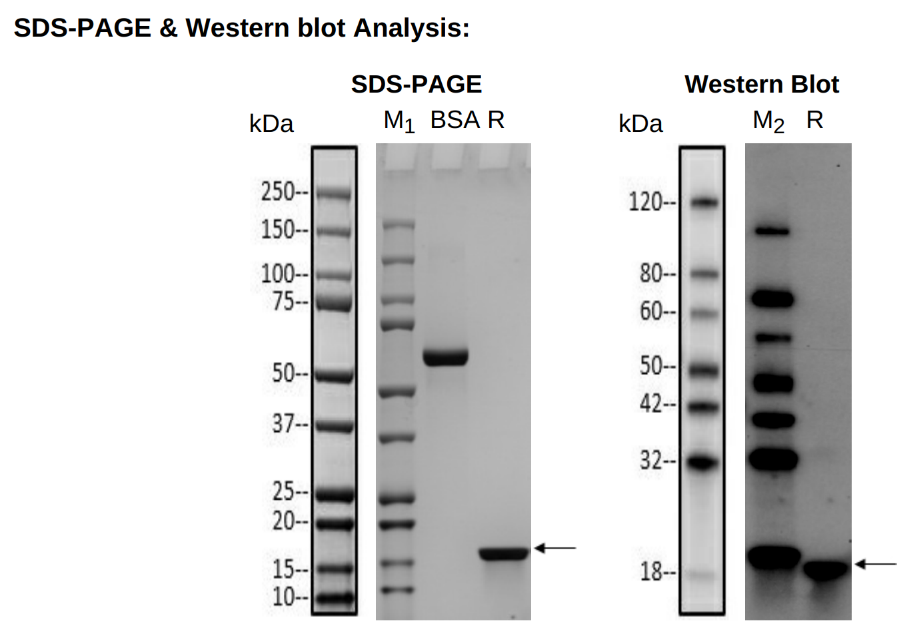


Fig.S8 SDS-PAGE and Western blot validation of recombinant B2URF3 expression.

SDS-PAGE (left) and Western blot (right) were performed under reducing conditions. A single distinct band located at approximately 18–20 kDa (arrow) corresponds to the predicted molecular weight of His-tagged B2URF3, confirming its successful expression and purification. Anti-His monoclonal antibody was used for Western blot detection.

Lane M1: Protein Marker, Bio-rad, Cat. No. 1610374S, refer to annotated key on the left for size

Lane M2: Protein Marker, GenScript, Cat. No. M00673, refer to annotated key on the left for size

BSA: 2.00 μg

R: Reducing condition

Primary antibody: Mouse-anti-His mAb (GenScript, Cat.No. A00186)


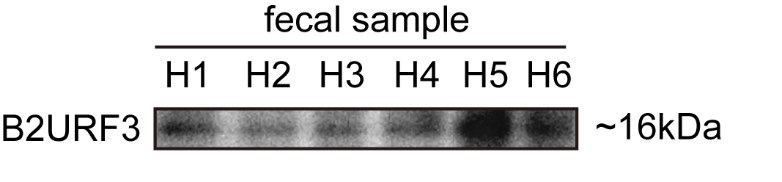


Fig.S9 Detection of B2URF3 protein in healthy human fecal-derived bacterial extracts.


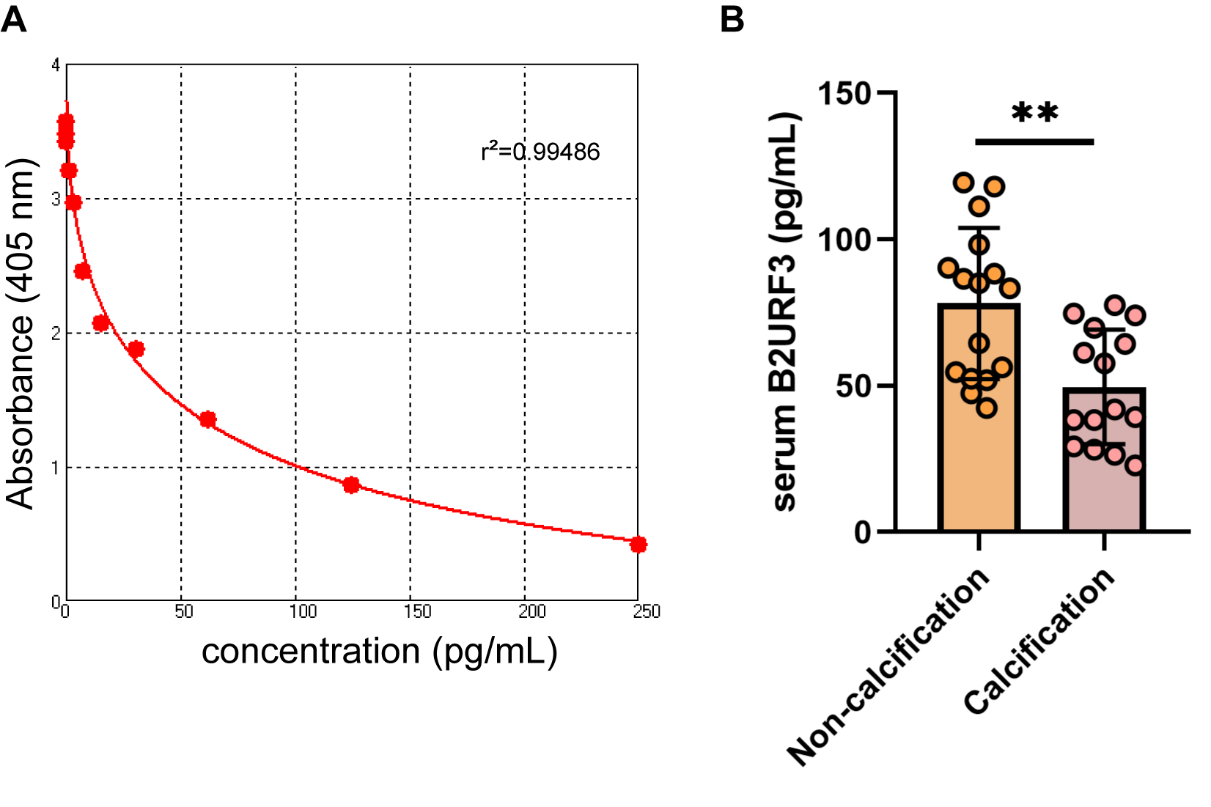


Fig.S10 ELISA analysis of B2URF3 levels in serum samples from patients with coronary artery calcification and control subjects. (A), Corresponding calibration curve of competitive ELISA. (B), serum B2URF3 concentration. ***P* < 0.01.


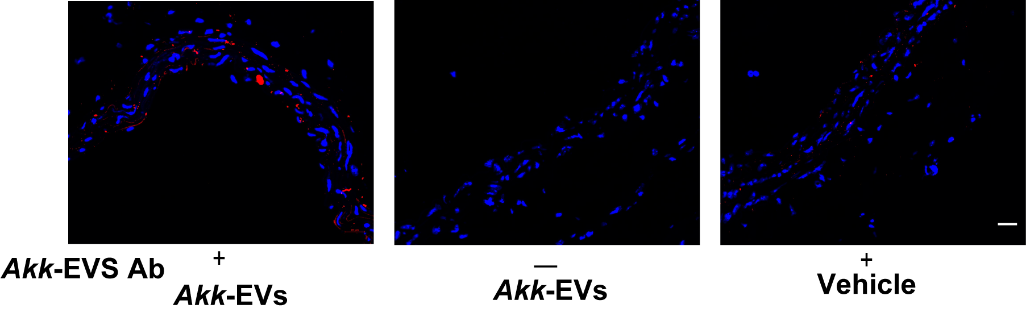


Fig.S11 Representative images of the *Akk*-EVs antibody (Ab)-stained aortic sections. Scale bar = 20 μm.


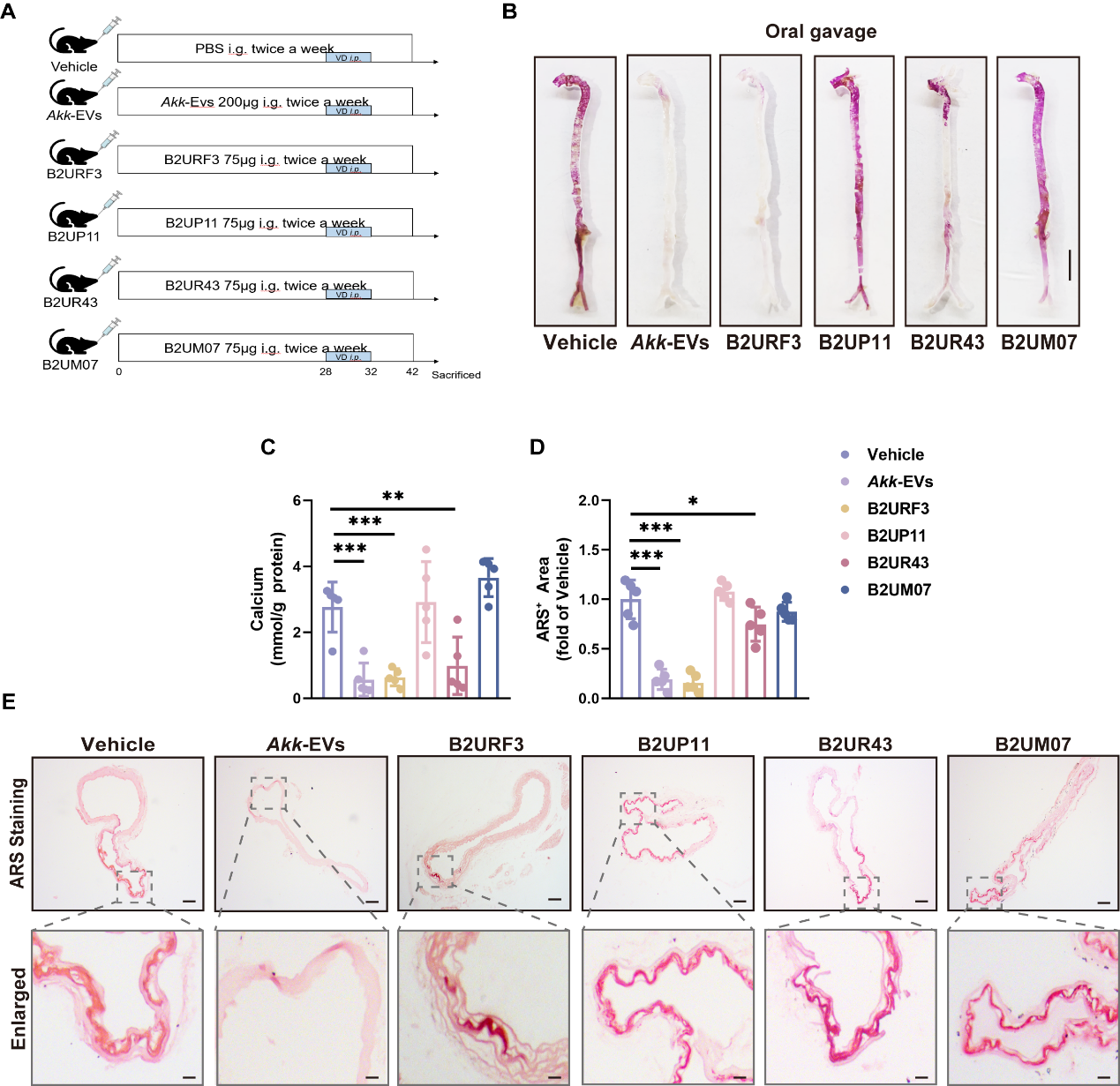


Fig.S12 Oral administration of *Akk*- EVs and B2URF3 attenuates vascular calcification in vivo.

(A), Schematic diagram of the experimental mechanism for oral gavage of *Akk*-EVs and uncharacterized proteins. (B), Representative image of the whole aorta stained with ARS. Scale bar = 5 mm. (C), Quantitative assessment of calcium content in the whole aorta. n = 5 for each group. (D), Quantification of ARS staining area in each group relative to the Vehicle group. n = 5 for each group. (E), ARS staining representative images of the aorta section. Scale bar = 100 μm (up) and 20 μm (bottom). Statistical significance was determined using one-way analysis of variance followed by Bonferroni *post hoc* test. Data are shown as mean ± SD. **P* < 0.05, ***P* < 0.01, ****P* < 0.001.


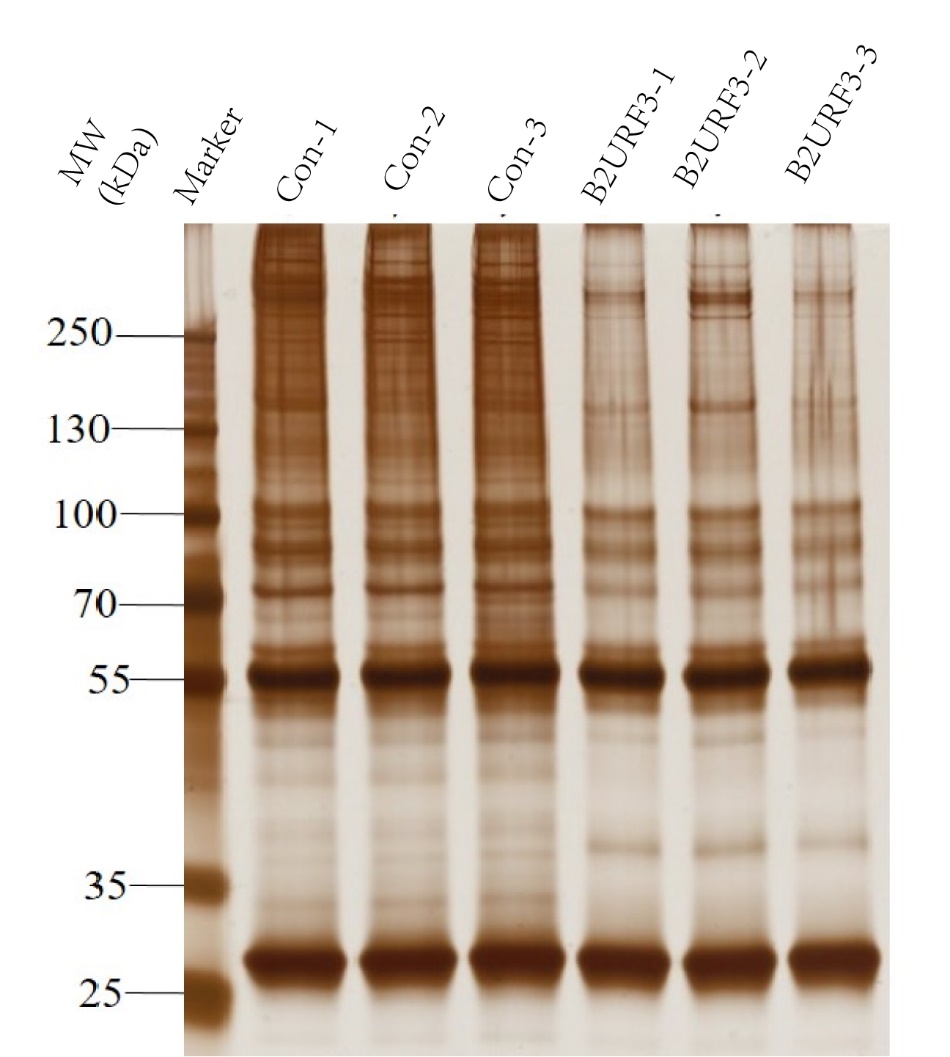


Fig.S13 Silver staining of proteins from IP-MS assay by the His-labeled B2URF3 probe in VSMCs. n = 3 for each group.


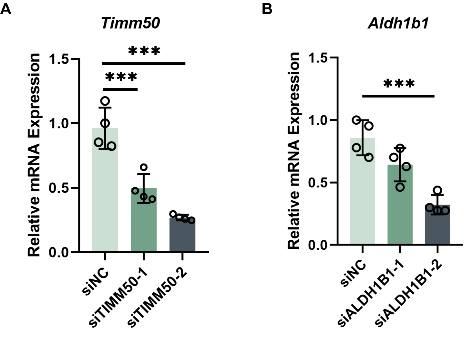


Fig.S14 Detection of the efficiency of TIMM50 (A) and ALDH1B1 (B) siRNA. n = 4 for each group. Statistical significance was determined using one-way analysis of variance followed by Bonferroni post hoc test. Data are presented as mean ± SD. ****P* < 0.001.


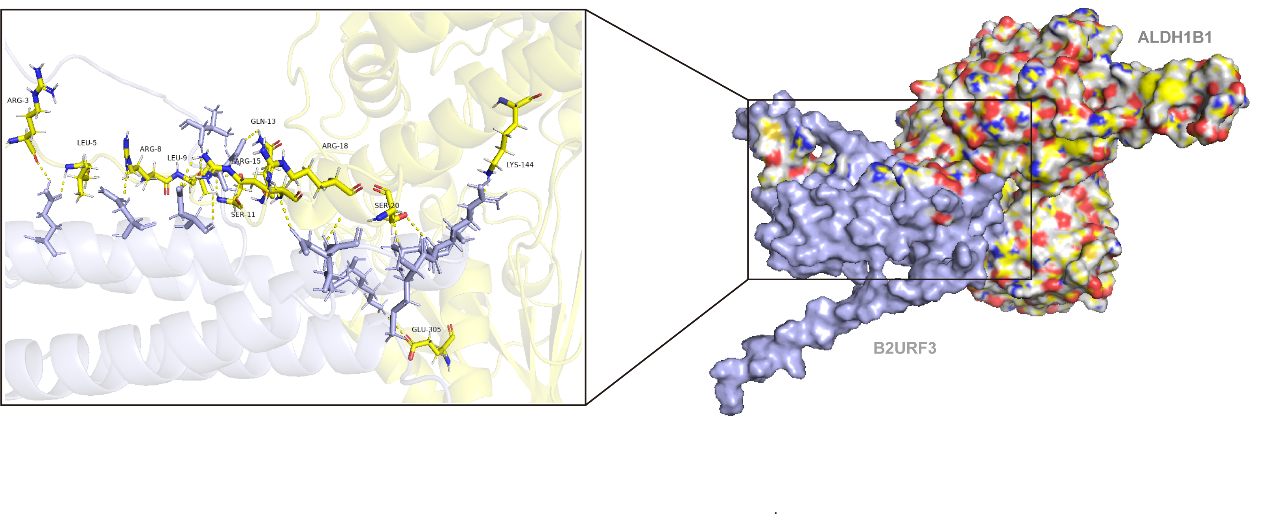


Fig.S15 The molecular docking between B2URF3 and ALDH1B1.

Table.S1 Clinical characteristics of calcification patients and no calcification disease patients

whose fecal sample were collected.

| Variables | Non-calcification (n = 27) | Calcification (n = 27) | *P* value |
| --- | --- | --- | --- |
| Age, year  Men, n (%)  BMI, kg/m2  SBP, mmHg  DBP, mmHg  Diabetes, n (%)  Smoker, n (%)  Biochemistry | 54.93 ± 9.87  18 (66.67)  24.06 ± 4.115  133.7 ± 18.89  83.33 ± 11.99  3 (11.11）  8 (29.63) | 70.81 ± 8.93  17 (62.96)  22.36 ± 3.387  130.7 ± 19.71  73.74 ± 12.29  7 (25.93)  7 (25.93) | < 0.0001  0.7757  0.1103  0.5704  0.0059  0.2935  0.7613 |
| Fasting glucose, mmol/L  Total cholesterol, mmol/L  UA, μmol/L  K, mmol/L  Ca, mmol/L  Na, mmol/L | 4.77 (3.89–5.63)  4.42 (4.15–4.95)  352.2 ± 130.6  3.88 (3.61–4.08)  2.26 (2.18–2.32)  141.5 (139.3-142.7) | 4.87 (4.03–5.36)  3.93 (3.69–4.74)  394.8 ± 120.7  3.66 (3.41 ± 4.03)  2.24 (2.16–2.30)  141.3 (139.3-143.1) | 0.9624  0.086  0.2186  0.2724  0.5561  0.7476 |

Values are expressed as mean ± SD or median (25th to 75th quartiles) for continuous variables and n (%) for categorical variables, respectively. For continuous variables, statistical significance was assessed using Student’s t test or nonparametric Mann Whitney U test. For categorical variables, statistical significance was evaluated by χ2, or Fisher exact test. BMI, body mass index; SBP, systolic blood pressure; DBP, diastolic blood pressure; LDL-C, low-density lipoprotein cholesterol; CREA, creatinine; UA, uric acid; K, potassium; Ca, calcium; Na, sodium.

Table.S2 Clinical characteristics of calcification patients and no calcification disease patients

whose serum sample were collected.

| Variables | Non-calcification (n = 16) | Calcification (n = 15) | *P* value |
| --- | --- | --- | --- |
| Age, year  Men, n (%)  BMI, kg/m2  Abdominal circumference, cm  Smoker, n (%)  Biochemistry | 49.06 (43.25–60.25)  11 (68.75%)  22.59 (19.15–26.12)  84.42 ± 8.908  5 (68.75%) | 57.27 (55.00–63.00)  8 (53.33%)  25.2 (23.18–27.25)  90.38 ± 10.04  5 (66.67%) | 0.0528  0.3785  0.0704  0.1308  0.9013 |
| HbA1C, %  Total cholesterol, mmol/L  LDL-C, mmol/L  CREA, μmol/L  UA, μmol/L  K, mmol/L  Ca, mmol/L  Na, mmol/L | 10.09 ± 2.555  4.505 ± 1.407  2.785 ± 1.132  78.44 (53.25–102.30)  319.8 ± 103.1  3.994 ± 0.3327  2.198 ± 0.05811  137.1 ± 4.232 | 10.24 ± 2.941  4.923 ± 1.267  3.074 ± 0.994  89.67 (61.45–118.40)  336 ± 129.50  4.065 ± 0.3259  2.266 ± 0.1192  139.4 ± 0.8418 | 0.8838  0.3934  0.4574  0.7357  0.7058  0.5537  0.0509  0.1082 |

Values are expressed as mean ± SD or median (25th to 75th quartiles) for continuous variables and n (%) for categorical variables, respectively. For continuous variables, statistical significance was assessed using Student’s t test or nonparametric Mann Whitney U test. For categorical variables, statistical significance was evaluated by χ2, or Fisher exact test. BMI, body mass index; AC, abdominal circumference; HbA1C, Hemoglobin A1C; LDL-C, low-density lipoprotein cholesterol; CREA, creatinine; UA, uric acid; K, potassium; Ca, calcium; Na, sodium.
